# SELF-Tree: An Interpretable Model for Multivariate Causal Direction Heterogeneity Analysis

**DOI:** 10.1017/psy.2025.10067

**Published:** 2025-12-10

**Authors:** Zhifei Li, Hongbo Wen

**Affiliations:** Collaborative Innovation Center of Assessment for Basic Education Quality, Beijing Normal University, China

**Keywords:** causal discovery, heterogeneous causal direction, recursive partitioning method, structural equation likelihood framework

## Abstract

Identifying causal directions among variables via data-driven approaches is a research hotspot. Researchers now focus on detecting causal direction heterogeneity among multiple variables (variables more than two) when covariates cause such heterogeneity. This study combines the structural equation likelihood function (SELF) method with a recursive partitioning method to achieve an interpretable model of multivariate causal direction heterogeneity in multivariable settings. Through simulation, we compared the performance of the SELF-Tree model in terms of the identification about heterogeneous causal direction under different conditions. Using a public drug consumption dataset, we demonstrated its real data application. The SELF-Tree model offers researchers a new way to understand variable causal direction heterogeneity.

## 1 Introduction

Investigating causal relationships among variables is a fundamental goal of psychological research. By establishing causality, researchers can uncover the mechanisms that govern human behavior and design effective interventions to change it (Vowels, 2025). When variable X is the cause and variable Y the effect, we denote this causal relation as X→Y . For decades, randomized controlled trials (RCTs) have been regarded as the gold standard for causal inference because they systematically manipulate the putative cause and observe subsequent changes in the outcome. However, ethical constraints and practical costs often render RCTs infeasible, making the extraction of causal information from nonexperimental data a pressing issue across economics (Angrist & Pischke, 2010), sociology (Brand et al., 2023), education (Cordero et al., 2018), computer science (Pearl, 2009), and psychology (Vowels, 2025). Current observational approaches fall into two broad categories: causal inference and causal discovery. Causal inference prespecifies the cause–effect pair and then estimates the true causal effect while adjusting for confounders (Schafer & Kang, 2008). Causal discovery, by contrast, leverages distributional properties to uncover the directionality of causal links directly from observational data (Glymour et al., 2019; Zanga et al., 2022), thereby offering an exploratory tool for theory construction (Vowels, 2025).

However, the causal relationships between psychological variables often exhibit a “chicken or egg” relationship in which the causal direction is ambiguous, such as pride and social rank (Witkower et al., 2022), work–family conflict and strain (Nohe et al., 2015), and job insecurity and mental health complaints (Griep et al., 2021). Some researchers treat these patterns as reciprocal relationships and analyze them with cross-lagged panel models (Zhang et al., 2025). Recent work, however, suggests that the underlying causal direction may be unidirectional but heterogeneous across individuals that only X→Y or Y→X within any given subgroup (Zhang & Wiedermann, 2024). Apparent reciprocity then arises from aggregating subpopulations with opposite directions—what we term heterogeneous causal directions. For example, X→Y may hold when Z≤0 multivariable contexts (e.g., De Clercq et al., 2013; Sheu et al., 2022), but tools that are simultaneously multivariate and interpretable remain scarce.

As many psychological theories posit unidirectional links (Zhang et al., 2025), providing quantitative evidence for a specific causal direction—even through exploratory methods—can advance theory development. Although most causal-discovery algorithms assume data homogeneity, interest in heterogeneous causal directions is growing. Early investigations appeared in genomics, where the causal order among genes varies across samples (Ni et al., 2018; Zhou et al., 2023). Social-science researchers have recently followed suit: Wiedermann and colleagues examined bidirectional heterogeneity between two variables (e.g., Li & Wiedermann, 2020; van Wie et al., 2019; Wiedermann et al., 2024), and neural-network approaches have uncovered multivariate heterogeneity under different conditions (Thompson et al., 2024). These models, however, are largely black boxes; they describe what heterogeneous patterns emerge but not why. Such opacity hampers psychological theory, which prizes interpretability.

Recursive partitioning offers a promising alternative. By recursively splitting data according to if-then rules, tree-based models capture how covariates modulate variable relationships, yielding transparent, rule-based subgroups (Strobl et al., 2009; Zeileis et al., 2008). Recursive partitioning has been integrated with numerous psychometric models to explore heterogeneous correlational patterns (Brandmaier et al., 2013; Fokkema et al., 2018; Fokkema & Zeileis, 2024; Jones et al., 2020; Kiefer et al., 2024). In causal inference, Bayesian additive regression trees (Chipman et al., 2010; Hill, 2011) and causal trees (Athey & Imbens, 2016) successfully detect heterogeneous treatment effects. In causal discovery, recursive partitioning has also recently been used to identify heterogeneous causal directions between two variables (Wiedermann et al., 2024). Yet its application to multivariate heterogeneous causal directions remains largely unexplored.

The present study bridges this gap by combining causal-discovery techniques with tree-based recursive partitioning to identify and interpret multivariate heterogeneous causal directions. Specifically, we employ conditional inference trees (CTree; Hothorn, Hornik, & Zeileis, 2006)—a nonparametric tree algorithm—to partition participants into homogeneous subgroups defined by covariates. Within each subgroup, we apply the Structural Equation Likelihood Function (SELF; Cai et al., 2018), a state-of-the-art causal-discovery method, to determine the causal ordering of multiple variables. We call this integrated approach the SELF-Tree method. To our knowledge, this is the first attempt to introduce recursive partitioning into the discovery of multivariate heterogeneous causal directions.

We first review relevant literature on causal discovery, heterogeneous causal directions, and recursive partitioning. We then formalize the SELF-Tree framework and evaluate its performance under various conditions via simulation. Finally, we provide an empirical illustration to demonstrate its practical utility. Through these steps, we highlight how marrying causal discovery with tree-based partitioning can yield an interpretable, exploratory tool for uncovering multivariate heterogeneous causal structures, and we document the effectiveness of the SELF-Tree method.

## 2 Literature review

### 2.1 Causal discovery

**Figure 1:**
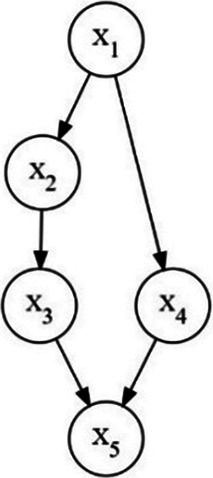
An example of a directed acyclic graph.

In the causal-discovery framework, causal relationships are represented as directed acyclic graphs (DAGs; Greenland et al., 1999; Morgan & Winship, 2014), also termed causal graphs. Let $X={\left(X_{1},\cdots ,X_{p}\right)}^{T}$ denote the p random variables. The DAG encoding their causal relationships is $G=\left(V,E\right)$ , where $V=\left.l,b,r,a,ce1,\cdots ,p,r,b,r,a\right.ce$ indexes the nodes and node and i corresponds one-to-one of variable $X_{i}\;\left(1\leq i\leq p\right)$ ; in what follows, we sometimes refer to variable $X_{i}$ and its corresponding node i interchangeably. E denotes the directed edges, which reflect the causal–effect relationships between variables. The nodes and directed edges are the two fundamental components of a DAG. Figure 1 provides an example: $X_{1}\to X_{2}$ indicates that $X_{1}$ exerts a direct causal influence on $X_{2}$ , whereas the absence of the directed edge between $X_{3}$ and $X_{4}$ implies no direct causal link. DAGs forbid simultaneous causation; only unidirectional edges are permitted, ruling out bidirected edges (e.g., $X_{1}\to X_{2}\to X_{1}$ ) or cycles (e.g., $X_{1}\to X_{2}\to X_{3}\to X_{1}$ ).

A DAG describes the data-generating process. For any i≠j∈V , i is the parent node of j if and only if $X_{i}\to X_{j}$ , and $PA_{G}\left(X_{j}\right)$ represents the set of all parent nodes of $X_{j}$ in G . The causal-discovery algorithms infer the true causal graph G from an observed dataset D . Two variables are connected by a *path* if they are linked through a sequence of nodes and directed edges. The path does not require the directed edges with the same direction. For instance, in Figure 1, $X_{3}\leftarrow X_{2}\leftarrow X_{1}\to X_{4}$ constitutes a path between $X_{3}$ and $X_{4}$ .

To recover causal relations from data, three standard assumptions are usually invoked: causal sufficiency, the causal Markov property, and causal faithfulness (Glymour et al., 2019; Zanga et al., 2022; Zhou et al., 2023). The causal sufficiency states that every common cause of variables in the graph has been measured. The causal Markov property and causal faithfulness are complementary. The causal Markov property states that conditional independences in the joint probability distribution of the variables can be inferred from the d-separation in the causal graph G , whereas causal faithfulness asserts that every conditional independence found in the distribution corresponds to a d-separation in the graph (Pearl, 2009). Under these two assumptions, d-separations in G are in one-to-one correspondence with conditional independencies in the joint probability distribution. When the assumptions hold, the joint distribution of the variables in G factorizes as follows:

(1) $$\begin{array}{c} \mathrm{Pr}\left(X\right)=\prod_{j=1}^{p}\mathrm{Pr}\left(X_{j}|\left.l,b,r,a,ceX_{i}:i\in PA_{G}\left(X_{j}\right),r,b,r,a\right.ce\right) \end{array}$$

where $\mathrm{Pr}\left(X_{j}|\left.l,b,r,a,ceX_{i}:i\in PA_{G}\left(X_{i}\right),r,b,r,a\right.ce\right)$ denotes the conditional probability distribution of $X_{j}$ given its parent nodes. Combining with the structure of G , it yields the corresponding joint probability distribution of variables, which is symbolled as F (Zanga et al., 2022; Zhou et al., 2023). Two DAGs, G and $G^{\prime }$ , are said to belong to the same Markov equivalence class (MEC), if they generate probability distributions, F and $F^{\prime }$ , admit identical factorizations (Andersson et al., 1997). For example, both $X_{i}\leftarrow X_{j}\to X_{k}$ and $X_{i}\to X_{j}\to X_{k}$ imply the same factorization $\mathrm{Pr}\left(X_{i},X_{j},X_{k}\right)=\mathrm{Pr}\left(X_{j}\right)\times \mathrm{Pr}\left(X_{i}\right|X_{j})\times \mathrm{Pr}\left(X_{k}\right|X_{j})$ , even though the causal relationships between $X_{i}$ and $X_{k}$ are totally different in the two graphs^1^.

Current causal-discovery methods fall into three broad classes: constraint-based, score-based, and distributional-asymmetry-based (Vowels, 2025). Constraint- and score-based methods, often referred to collectively as global view methods (Cai et al., 2018), focus primarily on settings with more than two variables. Constraint-based approaches identify causal relations by testing conditional independencies; under the faithfulness assumption, these independencies are mapped onto graphical properties to yield an estimated $\hat{G}$ . Representative algorithms include the Peter–Clark algorithm (Spirtes et al., 2000) and inductive causation (Pearl & Verma, 1995). Score-based methods search for the DAG that maximizes a scoring function $S\left(G,D\right)$ , i.e., $\hat{G}={argmax}_{G\in G}S\left(G,D\right)$ , where G is the set of all possible DAGs over the given nodes; the greedy equivalence search (Alonso-Barba et al., 2013) is a well-known example. A key limitation of global-view methods is that they typically recover only a completed partially directed acyclic graph (CPDAG; Chickering, 2003) rather than a unique DAG, because they cannot fully resolve the Markov equivalence classes.

Distributional-asymmetry-based methods, also called local view methods (Cai et al., 2018), focus on bivariate settings. They exploit nonlinearity or non-Gaussianity—features that produce structural asymmetries—to determine causal direction. Representative techniques include the linear non-Gaussian acyclic model (Shimizu et al., 2006, 2011), additive noise models (Peters et al., 2014), post-nonlinear models (Zhang et al., 2016), information geometric models (Janzing et al., 2012), and direction dependence analysis (DDA; Wiedermann, 2022).

Recognizing that each class of methods has distinct strengths and weaknesses, researchers have begun to combine them. The max-min hill-climbing (MMHC) algorithm (Tsamardinos et al., 2006) first uses a constraint-based step to learn the skeleton of the graph and then employs a score-based step to orient the edges. Similar mixed-method approaches include the greedy fast causal inference method (Ogarrio et al., 2016) and the scalable causation discovery algorithm (Cai et al., 2013). A more recent example is the SELF algorithm (Cai et al., 2018), which integrates score-based global optimization with distributional-asymmetry-based local decisions to yield a globally coherent DAG (rather than a CPDAG) while retaining the statistical rigor of local view methods (Chen et al., 2022). The local view methods provide sharper insight into the causal direction between variable pairs, thereby avoiding the MEC in which the orientation can no longer be determined, whereas global view methods assemble local information to select the most plausible overall causal-graph structure and correct possible errors committed at the local level. The SELF algorithm yields a globally optimized model that retains local statistical significance and delivers a deterministic, theoretically robust decision on causal direction by integrating these two ideas (Cai et al., 2018). The further algorithmic details of SELF are given in Section 3.2.

### 2.2 Identification for heterogeneous causal directionality

In traditional causal-discovery analysis, the data is often assumed to be independent and identically distributed (Cai et al., 2018; Pearl, 2009). However, the real-world samples may not meet this assumption (Ickstadt et al., 2011; Oates et al., 2016). If ignored, the overall model’s causal directions may greatly differ from the true ones (Thompson et al., 2024). Researchers commonly use the term heterogeneous causal structure learning to capture the possibility that complex data sets exhibit causal relationships that differ across subpopulations (Zhou et al., 2025). Because participants may be drawn from distinct contexts—such as different geographic regions or time periods—their lifestyles often vary substantially. Such contextual differences can induce data heterogeneity: across subgroups, the distribution of noise variables, the magnitude of causal effects, and even the direction of causal relationships may all change (Zhou et al., 2025).

Consequently, researchers have developed new causal-discovery methods to identify heterogeneous causal directions and obtain heterogeneous DAGs. These DAGs involve the same variables but different causal directions (i.e., directed edges). Early researchers focused on identifying heterogeneous DAGs directly from variables when the number of heterogeneous DAGs was known (e.g., Oates et al., 2016; Yajima et al., 2015) or unknown (e.g., Ickstadt et al., 2011). The heterogeneous reciprocal graphical models (hRGMs) offer a unified framework for analyzing heterogeneous DAGs, covering both known and unknown situations (Ni et al., 2018). Löwe et al. (2022) introduced an amortized causal-discovery framework that leverages neural-network models and temporal information to identify distinct causal graphs.

**Figure 2:**
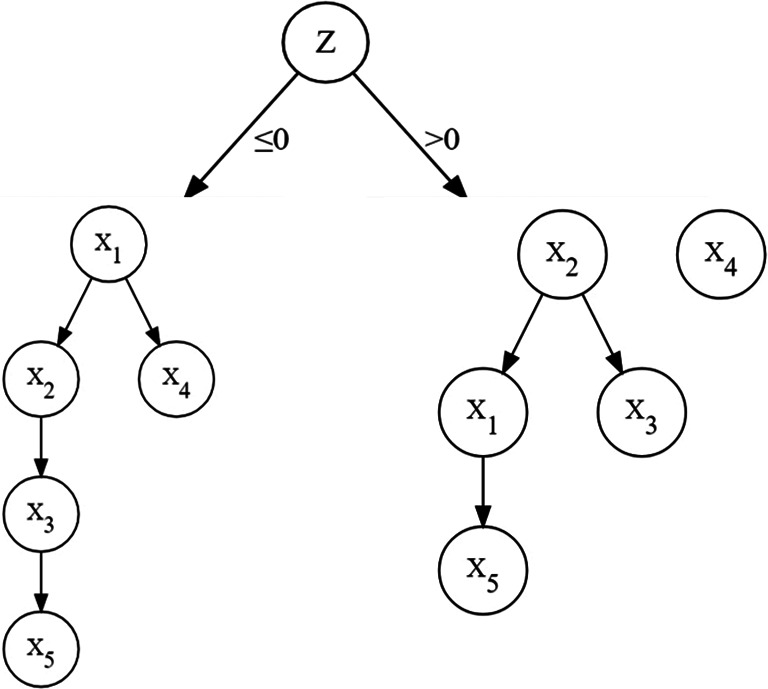
Causal direction heterogeneity of variables under different covariate values. Figure 2 long description.

Recent studies have focused on how exogenous covariates affect causal directions (Ni et al., 2019; Thompson et al., 2024; Zhou et al., 2023). Figure 2 shows causal direction heterogeneity under different values of covariate Z . Researchers proposed to incorporate covariates into the heterogeneous DAGs identification. For instance, Ni et al. (2019) developed the Bayesian graphical regression (BGR) method, which allows the directed graph to change with multiple covariates, whether continuous, discrete, or a mix. BGR captures nonlinear relationships between DAGs and covariates using smooth and thresholding functions, enabling smooth DAG structure changes with covariate values. To ensure the effective identification of heterogeneous DAGs, BGR imposes sparsity constraints on directed edges based on a unified DAG.

A similar Bayesian approach for heterogeneous causal direction identification is the latent trajectory embedded Bayesian network (BN-LTE) method (Zhou et al., 2023). It aggregates multiple covariates into a continuous latent variable (referred to as the *pseudotime* variable) and identifies heterogeneous causal directions based on the covariation between the latent variable and the DAG structure. The latent variable can be seen as a reordering of all covariates, enabling the DAG structure to change smoothly with it. Additionally, based on the latent variable, the BN-LTE method can identify certain Markov equivalence classes, aiding in clarifying the causal directions among variables.

Recently, the neural network algorithm has been used to identify the heterogeneous causal directions. Thompson et al. (2024) built a covariate-based feature space into a neural network to recognize heterogeneous DAGs with reframing the DAG structure learning problem as a continuous optimization problem. They added a projection layer to ensure the acyclic characterization of the causal graph structure (Bello et al., 2022). Unlike Ni et al. (2019), the neural network algorithm can directly learn node order from data, needing no prior assumptions. Using the neural network algorithm, Thompson et al. (2024) studied the DAG differences of 18 recreational drug use scenarios under low- and high-scoring conditions of neuroticism and sensation-seeking.

### 2.3 Recursive Partitioning Method

The recursive partitioning method, also known as the tree model, is a data-driven tool for identifying differential variable relationships and how covariates influence them (Strobl et al., 2009). These differential relationships include heterogeneous causal relationships (e.g., Athey & Imbens, 2016; Hill, 2011) and broader variable correlations differing under various covariates, i.e., the interaction terms of variables and covariates (Zeileis et al., 2008). The recursive partitioning method used hierarchical if-then rules to partition samples into categories, forming a tree structure T (Breiman et al., 1984). These rules are mutually exclusive and exhaustive, automatically generated by learning algorithms. In practice, researchers form the if-then rules based on their optimization procedure to split the values of covariates.

The *tree-nodes* ^2^ are employed for representing heterogeneous variable relationships in the recursive partitioning method, including the root-node, internal-nodes, and leaf-nodes. The if-then rules form paths from the root-node to leaf-nodes, with internal-nodes representing rule conditions and leaf nodes indicating classification results. It allows for rich variable information visualization. Figure 2 illustrates how the recursive partitioning method shows the impact of covariates on causal directions between variables. A key focus in tree model construction is selecting covariates and split points at specific tree-nodes. Current approaches include model-based partitioning (MOB; Zeileis et al., 2008) and CTree (Hothorn, Hornik, & Zeileis, 2006). MOB, a semiparametric method, assumes variables follow different probability distributions (often Gaussian) under different covariate conditions and uses log-likelihood functions to determine split points. CTree, a nonparametric recursive partitioning method, employs permutation test (Hothorn, Hornik, van de Wiel, et al., 2006; Strasser & Weber, 1999) to catch the statistics capturing relationships between variables and covariates.

The recursive partitioning method has been utilized for identifying heterogeneous variable relationships. These models are applied to explore and explain the parameterization differences with the combination of some psychological statistics and measurement models, such as structural equation model (Brandmaier et al., 2013; Kiefer et al., 2024), network analysis model (Jones et al., 2020), and generalized linear mixed model (Fokkema et al., 2018; Fokkema & Zeileis, 2024). In causal inference research, methods like Bayesian additive regression trees (BART, Chipman et al., 2010; Hill, 2011) and causal trees (Athey & Imbens, 2016) also highlight the importance of recursive partitioning method in calculating heterogeneous treatment effects.

To get more interpretable heterogeneous causal direction identification results, some researchers have applied the recursive partitioning method to identify causal direction heterogeneity in two variable cases (Li & Wiedermann, 2020; van Wie et al., 2019; Wiedermann et al., 2024). These studies mainly use the DDA to identify causal directions between variables. The DDA approach identifies the causal direction between two non-Gaussian variables by exploiting the higher-order moments of the predictor and the residuals of the causally competing models (Chen & Chan, 2013; Wiedermann, 2018, 2022), or the independence properties between predictor and residual (Shi et al., 2023; Wiedermann et al., 2024). When combined with recursive partitioning, DDA can leverage covariate information to distinguish a direct causal link ( X→Y ) from an unmeasured-confounder structure ( X←Confounders→Y ) (van Wie et al., 2019), or to pinpoint the specific causal direction in heterogeneous subgroups ( X→Y vs. X←Y ) (Wiedermann et al., 2024). Using tree-based models, Wiedermann and colleagues further examined the heterogeneous causal directions between *analog magnitude code* and *auditory verbal code*.

### 2.4 Summary

The use of causal-discovery methods to explore and identify causal relationships—especially causal direction—has attracted growing attention (Glymour et al., 2019; Vowels, 2025; Zanga et al., 2022). Recent studies increasingly emphasize heterogeneous causal directions and examine how covariate values shape these directions, leading to a family of methods that account for such heterogeneity. Yet these approaches suffer from two major limitations. First, although they can detect heterogeneous causal directions, they rarely scrutinize how participants are partitioned; this oversight can foster misinterpretation. As illustrated in Figure 2, the causal direction between two variables may conflict across conditions. Pooling participants without acknowledging these differences risks implying a cyclic relationship, thereby violating the acyclicity assumption of DAGs. Second, most existing methods focus solely on identifying causal directions and provide little insight into why heterogeneity arises—that is, under which covariate configurations a specific causal direction emerges. For example, the BGR requires a general directed acyclic graph to identify the influence of covariates on causal relationships, and its predictive effectiveness largely depends on whether the general DAG can reflect all possible variable relationships. The BN-LTE method needs to aggregate information from multiple covariates to identify heterogeneous DAGs and has difficulty isolating the impact of an individual covariate. Neural network methods can effectively identify changes in DAGs with multiple covariates but still rely on researchers’ subjective judgment to determine the heterogeneity of causal directions. Nowadays, the recursive partitioning method offers an effective way to identify the influence of covariates on heterogeneous causal directions (Li & Wiedermann, 2020; van Wie et al., 2019; Wiedermann et al., 2024); however, it has not been applied to the identification and explanation of heterogeneous DAGs among multiple variables.

## 3 Linking causal discovery with recursive partitioning

In this section, we elaborate our conceptualization of heterogeneous causal directions and present a corresponding solution. Considering the SELF’s demonstrated capacity to integrate global- and local-view perspectives into a deterministic and statistically robust causal-discovery result (Cai et al., 2018), we adopt it as a benchmark causal-discovery method and embed it within a recursive partitioning framework to produce an interpretable model of heterogeneous causal directions. As SELF is a nonparametric method, we specifically employ the conditional inference tree (Hothorn, Hornik, & Zeileis, 2006) to couple causal discovery with recursive partitioning, thereby explaining how to identify heterogeneous DAGs and, crucially, how to interpret their origins. Our exposition proceeds in two stages. First, we outline the theoretical underpinnings of heterogeneous causal directions by focusing on what we term the “bidirectional relationship” among variables, thereby clarifying how such heterogeneity can be understood at an aggregate level. Second, we detail the construction of the SELF-Tree model—an integrative framework that combines SELF with CTree—to operationalize our approach to detecting and explaining heterogeneous causal directions.

### 3.1 Conceptual illustration

Our understanding of heterogeneous causal directions is rooted in an explanation of the “chicken-or-egg” relationship—also framed as bidirectional or seemingly cyclical causation—of variables. In the formal frameworks of causal inference and causal discovery, acyclicity is a cornerstone assumption that guarantees model identifiability. Empirical reality, however, abounds with examples of mutual reinforcement between two constructs (e.g., Lu et al., 2020; Nohe et al., 2015). In large-scale cross-sectional surveys, the likelihood that any variable pair exhibits a chicken-or-egg pattern increases rapidly as more variables are included. Traditional causal-discovery algorithms, designed for strictly unidirectional and acyclic relations, struggle to accommodate such reciprocity.

Time-series designs can disentangle reciprocal effects: X at time=t may influence Y at time=t+1 , while Y at time=t simultaneously influences X at time=t+1 (Löwe et al., 2022). Yet most current causal-discovery techniques remain focused on cross-sectional data (e.g., Cai et al., 2018; Janzing et al., 2012; Shimizu et al., 2006; Wiedermann et al., 2024). We therefore propose an alternative lens: any single time-point data set is a mixture of distinct participant subpopulations, each characterized by its own causal direction between the variables. When these subgroup differences are ignored, the aggregate pattern misleadingly appears as a chicken-or-egg loop.

Viewing the problem through heterogeneous causal directions can resolve this paradox, even if the conclusion is counter-intuitive. Consider the relationship between social reality and stereotypes. Early work argued that stereotypes arise endogenously from people’s perceptions of real group characteristics (Ford & Stangor, 1992). Conversely, stereotypes can also be shaped by exogenous cues such as social categorization and meaning categories, subsequently creating a discriminatory social reality. Leveraging the recent popularity of Western astrological signs in China, Lu et al. (2020) showed that the stereotype “Virgos are disagreeable—compulsive and nit-picking” generated a real social pattern of avoiding Virgos as colleagues or friends. In other words, the causal arrow between social reality and stereotypes flips depending on contextual covariates such as geographic origin, sampling time, or community setting. Where stereotypes originate, *social reality* → *stereotypes*; where they merely spread, *social categories* → *stereotypes* → *social reality*.

We regard this perspective not as a refutation but as a complement to the concept of reciprocal causation. Reciprocal accounts posit that X influences Y and Y subsequently influences X , a sequence that can be traced across multiple time points. We concur, with one caveat: at any single moment, the causal relation is unidirectional: either X→Y or Y→X , never both simultaneously. As Morgan and Winship (2014) emphasize, reciprocity does not imply simultaneous cause and effect; rather, the data at hand lack the temporal resolution to disentangle the sequence. Participants in a cross-sectional sample may be distributed across different stages of the same process. Whether they currently occupy the path *social reality* → *stereotypes* or *stereotypes* → *social reality* depends on covariate-defined subgroups.

Moreover, ignoring this heterogeneity compromises accuracy. Ni et al. (2019) demonstrated that a single DAG fitted to the full sample failed to capture subgroup-specific causal directions. Representing all subpopulations with one graph is therefore inadequate. Although longitudinal data would provide the clearest temporal ordering, cross-sectional data can still be salvaged: by partitioning participants on key covariates and estimating separate causal directions within each subgroup, we can both identify and explain heterogeneous causal structures. This insight reframes every cross-sectional causal-discovery study: whenever chicken-or-egg ambiguity arises, incorporating covariate information to delineate subpopulations offers a viable route to valid and interpretable causal inference.

### 3.2 The SELF-Tree model

We propose SELF-Tree, an interpretable algorithm for identifying heterogeneous causal directions. By integrating the strengths of the structural equation likelihood function—which accurately determines causal directions among multiple variables—and the CTree model. SELF-Tree both detects and explains multivariate causal heterogeneity. SELF incorporates noise-term information, thereby circumventing the MEC indeterminacy common to traditional approaches (Cai et al., 2018; Chen et al., 2022). Because SELF is nonparametric, we pair it with the nonparametric CTree model in the partitioning stage (Hothorn, Hornik, van de Wiel, et al., 2006; Jones et al., 2020).

Let p and q be the number of variables that constitute the causal DAG and covariates that may produce heterogeneous causal directions, respectively, denoted as $X={\left(X_{1},\cdots ,X_{p}\right)}^{T}$ and $Z={\left(Z_{1},\cdots ,Z_{q}\right)}^{T}$ . The study proposes a “two-step” approach for SELF-Tree.

**Step 1:** Using the CTree method to determine the tree structure T ;

**Step 2:** Constructs the SELF models for each leaf-node data to identify the causal direction among multiple variables, yielding a covariate-specific DAG $G\left(Z\right)$ together with its corresponding structure function $F\left(Z\right)$ .

Following the above steps, the final output is a tuple ⟪leT,G,F⟫le . In our design, the variables X and covariates Z are prespecified by the researcher. Crucially, the covariates Z are used solely for partitioning via CTree and never appear as nodes in the DAG; conversely, the variables X are employed exclusively to construct the DAG and play no role in the partitioning step. There is a detailed exposition of the CTree and SELF algorithms respectively at the following parts.

The CTree method employs permutation test to select the covariate $Z_{j}\;\left(1\leq j\leq q\right)$ at each tree-node that best explains heterogeneity. Following the network-tree framework proposed by Jones et al. (2020), we choose one covariate $Z_{j}$ at the current node and determine its optimal split point. Once the candidate covariate $Z_{j}$ is fixed for a given node, we construct a test statistic that quantifies the association between the variables X and the covariate $Z_{j}$ as follows:

(2) $$\begin{array}{c} T_{j}=vec\left(\sum_{i=1}^{n^{*}}g\left(Z_{ij}\right)h{\left(X_{i\cdot }^{*}\right)}^{T}\right) \end{array}$$

where $n^{*}$ is the number of participants in the tree-node, $X_{i\cdot }^{*}$ refers to the p -dimensional vector of standardized observations for i th participant at the current tree-node, the $g\left(\cdot \right)$ and $h\left(\cdot \right)$ are the transformation function for the covariate $Z_{j}$ and $X_{i\cdot }^{*}$ respectively. The transformation functions capture how $X_{i\cdot }^{*}$ changes with $Z_{j}$ . If $Z_{j}$ is numeric or ordinal, the $g\left(\cdot \right)$ is simply a scalar function; if $Z_{j}$ is nominal, a dummy covariate is created for each category, yielding a vector-valued $g\left(Z_{j}\right)$ . The $h\left(\cdot \right)$ is also called the influence function, which indicates the contribution of individual observations to the correlation between two variables. It can be expressed as

(3) $$\begin{array}{c} h\left(X_{i\cdot }^{*}\right)={\left(X_{i1}^{*}X_{i2}^{*},X_{i1}^{*}X_{i3}^{*}\cdots ,X_{i2}^{*}X_{i3}^{*},\cdots ,X_{i\left(p-1\right)}^{*}X_{ip}^{*}\right)}^{T} \end{array}$$

which only include the cross-products of the correlation. The means $X_{i1}^{*},\cdots ,X_{ip}^{*}$ and the squared standardized elements ${X_{i1}^{*}}^{2},\cdots ,{X_{ip}^{*}}^{2}$ can also be added in the influence function (Jones et al., 2020).

We apply the SELF model within each leaf-node to determine the causal directions among the variables. SELF assumes an additive-noise representation of the causal structure (Cai et al., 2018):

(4) $$\begin{array}{c} X_{j}=F_{j}\left(PA_{G}\left(X_{j}\right)\right)+E_{j} \end{array}$$

where $PA_{G}\left(X_{j}\right)$ denotes the parent set of $X_{j}$ in G , $F_{j}\left(\cdot \right)$ represents the structural equation, and $E_{j}$ represents the random noise term independent of $PA_{G}\left(X_{j}\right)$ . The error term $E_{j}$ is statistically independent of all variables X and other noise component. SELF requires that at least one of X or the noise vector $E={\left(E_{1},\cdots ,E_{p}\right)}^{T}$ exhibits distributional asymmetry (Janzing et al., 2012; Li & Wiedermann, 2020; Vowels, 2025; Wiedermann, 2022; Wiedermann et al., 2024), i.e., the relations among variables are nonlinear or the variables and the errors follow non-Gaussian distributions. Under linearity, the model simplifies to

(5) $$\begin{array}{c} X_{j}=\sum_{i=1}^{p}b_{ij}X_{i}+E_{j} \end{array}$$

with $b_{ij}$ representing the causal effect of $X_{i}$ on $X_{j}$ , $b_{ij}=0$ when $i\notin PA_{G}\left(X_{j}\right)$ . Note that under linearity and additive noise condition, at least one variable or error term must follow a non-Gaussian distribution. Let F collect all $F_{j}\left(\cdot \right)$ , and write S=<G,F>. Then the likelihood function over the variables by Equation (1) is

(6) $$\begin{array}{cc} L(S) & =\sum_{j=1}^{p}\log\left(\mathrm{Pr}\left(X_{j}=x|\left.l,b,r,a,ceX_{i}:i\in PA_{G}\left(X_{j}\right),r,b,r,a\right.ce\right)\right)\\ & =\sum_{j=1}^{p}\sum_{n=1}^{N}\log\left(\mathrm{Pr}\left(E_{j}=x-F_{j}\left(PA_{G}\left(X_{j}\right)\right)\right)\right) \end{array}$$

The SELF model maximizes a likelihood function constructed over the noise terms to obtain a globally optimal DAG while simultaneously ensuring the local statistical independence between the noise and the observed variables (Cai et al., 2018). The model places no restrictions on the data type—continuous or discrete—or on the functional form—linear or nonlinear—of the causal relationships. The DAG structure itself is estimated via a hill-climbing–based causal-structure search algorithm (Tsamardinos et al., 2006). The following theorems about the SELF model are established (Cai et al., 2018):

1. For observed data of X generated by causal structure S , the likelihood function satisfies $L(S)\geq L\left(S^{\prime }\right)$ for any other causal structure $S^{\prime }$ .
2. Under the assumption of independent and identically distributed observations, maximizing L(S) is equivalent to minimizing the sum of entropies $\sum_{j=1}^{p}H\left(E_{j}|S\right)$ , where $H\left(\cdot \right)$ denotes entropy. And if the correct parent nodes reduce entropy more than nonparental nodes, then the causal structure S that minimizes the entropy is the correct one.

By integrating the CTree and SELF frameworks, we obtain a procedure that identifies multivariate heterogeneous causal directions while explicitly accounting for covariate influences. For the SELF-Tree model to be valid, the following assumptions must hold:

**Assumption 1.** There exist distinct subpopulations whose causal directions among the variables are heterogeneous, and these subpopulations can be distinguished by the available covariates.

**Assumption 2.** The set of covariates is complete—i.e., all sources of heterogeneity are captured by the observed covariates.

**Assumption 3.** The covariates influence only the direction of the causal relations among the variables, not the variables’ values; thus, the DAG itself contains only the variables of interest.

**Assumption 4.** The variables and error terms exhibit distributional asymmetry, i.e., either nonlinear relationships among variables or non-Gaussian distributions of variables and noise.

**Assumption 5.** Within each leaf node obtained after conditioning on covariates, the data satisfy the causal sufficiency, the causal Markov property, and causal faithfulness; consequently, all observations in the same leaf share an identical causal direction, and the data are independent identically distributed. Across leaves, the estimated DAG structures may differ markedly.

In addition to the core assumptions outlined above, several auxiliary conditions are imposed to safeguard the validity of the SELF-Tree procedure: (i) all variables and covariates are free of measurement error (Gische & Voelkle, 2022; Wiedermann, 2022); (ii) no interference, i.e., the response of any unit is unaffected by the treatment assignment of other units (Rubin, 2005; Shin, 2012); and (iii) the covariates are mutually independent (Ni et al., 2019; Thompson et al., 2024). These restrictions preclude extraneous sources of bias and ensure that the identification performance of the SELF-Tree model is not confounded by additional error structures.

## 4 Simulation

### 4.1 Settings

We first evaluate the performance of SELF-Tree via simulation, focusing on (a) its ability to select relevant covariates and (b) its accuracy in recovering heterogeneous DAGs. Five factors are systematically manipulated:

1. Number of variables ( p ) in the true DAG.

2. Sample size ( N ).

3. Strength of causal relations, indexed by average indegree.

4. Mode of heterogeneity—i.e., how covariates shift causal directions.

5. Presence of spurious covariates.

**Figure 3:**
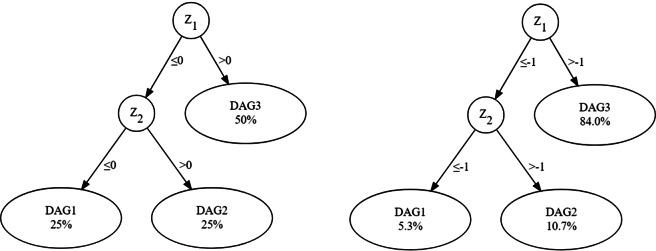
The tree structure in the simulation study. Note that the left is the *Moderate* structure, and the right is the *Extreme* structure. The percentage in each leaf node indicates the theoretical proportion of samples among all participants. The DAG1, DAG2, and DAG3 are used to label the causal graph structures under different conditions. Figure 3 long description.

For the first three factors, we set $N\in \left.l,b,r,a,ce500,1000,2000,4000,r,b,r,a\right.ce$ and $p\in \left.l,b,r,a,ce6,10,15,r,b,r,a\right.ce$ following Cai et al. (2018). The average indegree (AvgInd)—the mean number of incoming edges per node—captures inter-variable connectivity (Bloznelis, 2010). We vary $AvgInd\in \left.l,b,r,a,ce0.5,1.0,1.5,2.0,2.5,r,b,r,a\right.ce$ ; the lower bound p = 6 is chosen because 6 is the smallest number of variables that still permits a DAG when AvgInd = 2.5. Two covariates drive heterogeneity with $Z_{1}∼N\left(0,1\right)$ and $Z_{2}∼U\left(-3,3\right)$ . Both symmetric around 0. Two tree structures are examined as Figure 3 shows. The *Moderate* structure means that causal directions switch sharply at Z=0 , yielding balanced successor nodes. And the *Extreme* structure yielding the critical cut-point is at −1, producing highly unbalanced subsamples. Finally, two spurious covariates— and —are added to test whether redundant predictors degrade selection accuracy. All four covariates are mutually independent.

We use the *SELF* package (Cai et al., 2018) to generate the true causal graph and its corresponding data. In code, a directed graph is encoded as an adjacency matrix of size p×p . The element $\left(i,j\right)$ equals 1 if the edge $X_{i}\to X_{j}$ exists and 0 otherwise. The diagonal is necessarily 0. Owing to acyclicity, the matrix can always be written as an upper-triangular matrix. We therefore draw $\left(p^{2}-p\right)/2$ independent Bernoulli variables to fill the strictly upper-triangular positions with success probability $2\times AvgInd/\left(p-1\right)$ , which guarantees that the realized graph has an average indegree very close to the target value. A candidate matrix is accepted only if the total number of 1 s differs from p×AvgInd by no more than ±0.05. The accepted 1 s are then placed row-wise into the upper triangle to yield the true adjacency matrix. For data generation, we focus on linear non-Gaussian models, that is, the $X_{j}=\sum_{i=1}^{p}b_{ij}X_{i}+E_{j}$ , the $b_{ij}=0$ when $X_{i}\notin PA_{G}\left(X_{j}\right)$ . If the $PA_{G}\left(X_{j}\right)$ is empty, we set $E_{j}∼N\left(0,1\right)$ . Otherwise, for every parent $X_{i}\in PA_{G}\left(X_{j}\right)$ , we draw $b_{ij}∼U\left(-1,-0.5\right)\cup U\left(0.5,1\right)$ and $E_{j}$ to be a zero-mean noise with heavy tails and heteroskedastic variance ^3^.

### 4.2 Evaluation methods

The evaluation of the SELF-Tree model is divided into two parts: the recognition result of the tree structure and evaluation effect of the heterogeneous DAGs. The tree structure recognition includes the identification of the tree-nodes, that is, whether the positions of $Z_{1}$ and $Z_{2}$ align with those in Figure 3 specifically using the CTree model, and the accuracy of the split point estimation about every covariate using $Bias=1/n\;\sum_{i}\left|{\hat{z}}_{i}-z_{i}\right|$ , where n is the number of repetitions, $z_{i}$ is the true value of the split point in the simulation, and ${\hat{z}}_{i}$ is the estimated split point using the CTree method. A smaller bias value indicates a better estimation effect of the split point.

The evaluation of heterogeneous DAGs involves two aspects: the accuracy of the overall DAGs and the accuracy of directed edges in the DAGs. The overall accuracy of the DAGs is assessed using the structural Hamming distance (SHD) and the Frobenius norm (FNorm). SHD is the number of extra, missing, and reversed edges of the predicted directed acyclic graph $\hat{G}$ compared to the true graph G (Tsamardinos et al., 2006). FNorm measures the difference between the adjacency matrices of G and $\hat{G}$ (Shimizu et al., 2011). Smaller SHD and FNorm values indicate higher similarity between $\hat{G}$ and G . The accuracy of directed edge identification is evaluated using the true positive rate (TPR) and false positive rate (FPR; Cheng et al., 2022). Let $E_{G}$ be the edges in G and $E_{\hat{G}}$ be the edges predicted by $\hat{G}$ , we have

$$\begin{array}{c} TPR=\sum_{i}\frac{\#\left.l,b,r,a,cee_{i}|i\in E_{G}\cap E_{\hat{G}},r,b,r,a\right.ce}{\#\left.l,b,r,a,ceE_{G},r,b,r,a\right.ce},FRP=\sum_{i}\frac{\#\left.l,b,r,a,cee_{i}|i\in E_{\hat{G}}\backslash E_{G},r,b,r,a\right.ce}{\#\left.l,b,r,a,ceE\backslash E_{G},r,b,r,a\right.ce}. \end{array}$$

where E represents all possible edges in the directed acyclic graph, and $\#\left.l,b,r,a,ce\cdot ,r,b,r,a\right.ce$ counts the nonzero elements. The TPR measures the proportion of correctly identified edges, while FPR measures the proportion of incorrectly identified edges. Higher TPR and lower FPR values indicate better alignment between G and $\hat{G}$ . All these heterogeneous DAGs evaluation methods are based on the correct tree-node identification result.

### 4.3 Analysis procedure

This study uses the *partykit* package (Hothorn & Zeileis, 2015) in R language 4.4.2 (R Core Team, 2024) for nonparametric recursive partitioning with CTree models. It enhances the *SELF* package (Cai et al., 2018) for the structural equation likelihood framework method. The linear estimators and log-likelihood functions are employed to fit the SELF model.

### 4.4 Results

#### 4.4.1 The identification result of tree structure

**Figure 4:**
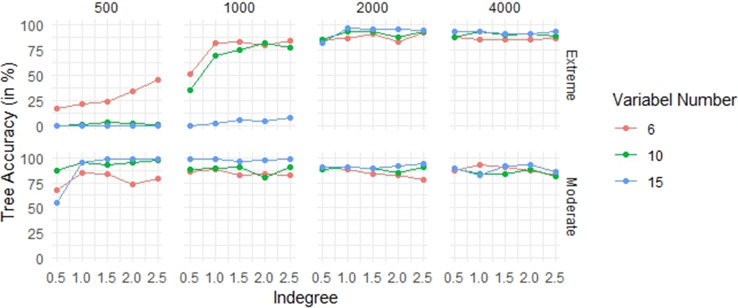
The identification result of tree structure. Figure 4 long description.

Figure 4 shows the accuracy of the tree-node identification under different conditions. In the *Extreme* structure, a sample size of over 2,000 can ensure a recognition accuracy of over 75%. The higher the indegree centrality of the DAGs, the better the accuracy of the tree-node identification. The impact of variables numbers on tree-node identification accuracy depends on the sample size: when the sample size is over 2,000, more variables can lead to better recognition, while fewer variables yield a better effect when the sample size is below 1,000. Notably, with a sample size of 500 and 15 variables (regardless of indegree centrality), and with a sample size of 1,000, an indegree centrality of 0.5, and 15 variables, the tree-nodes cannot be accurately identified with an *Extreme* tree structure. In the *Moderate* structure, except for a few cases, a recognition rate of over 80% can be generally ensured. Regardless of other conditions, an increase in the number of variables can improve the accuracy of tree-node identification.

The relationship between sample size, indegree centrality, and tree structure recognition accuracy is more complex: when the number of variables is 6, increasing the sample size can improve the tree-node identification accuracy, while increasing the indegree centrality reduces it; while when the number of variables is more than 10, increasing the sample size worsens the identification result, and the impact of indegree centrality becomes more complicated.

**Figure 5:**
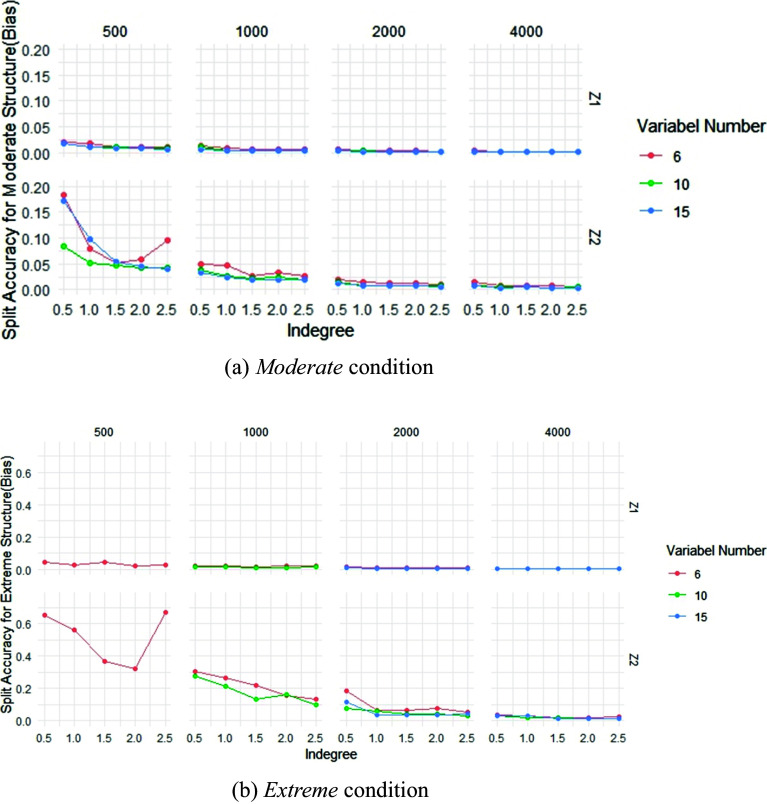
The identification result of the split point of the covariates. Note that under the *Extreme* condition, the tree model structure may not be accurately identified when the sample size is 500 with 10 or 15 variables, or when the sample size is 1,000 with 15 variables. Therefore, the split point identification result under these conditions is not presented in this figure. Figure 5 long description.

The results for the split point estimation of covariates are shown in Figure 5. The accuracy under the *Moderate* condition is significantly higher than the *Extreme* condition. The identification accuracy improves with increasing sample size and the number of variables. When the sample size is large (over 2,000), changes in indegree centrality do not significantly affect the split point identification accuracy. We also find that the identification accuracy for $Z_{1}$ (the covariate at the root node) is better than $Z_{2}$ , no matter what the tree structure is.

#### 4.4.2 The identification result of heterogeneous DAGs

**Figure 6:**
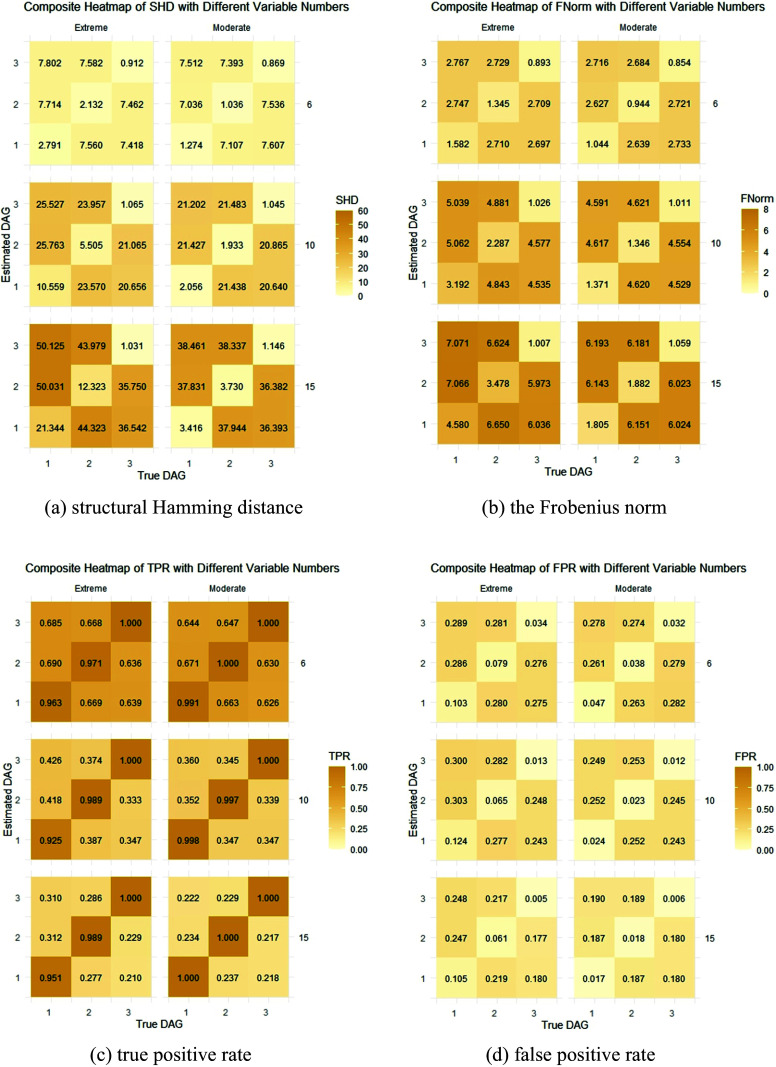
The impact of the number of variables on the identification of heterogeneous DAGs. Each 3 × 3 heatmap’s number label i corresponds to the DAG structure simulation scenario in Figure 3. The columns represent true DAGs, and rows represent the obtained DAGs based on the SELF-Tree model. Greater color contrast between the diagonal and off-diagonal areas of the heatmap indicates better recognition of heterogeneous causal directions. Figure 6 long description.

With the fixed sample size of 2,000 and the indegree centrality of DAG at 1.5, we evaluated the impact of the number of variables on the heterogeneous DAGs identification. The results are presented in Figure 6. Overall, the recognition effect of heterogeneous causal directions with *Moderate* structure is much better than the *Extreme* condition. Moreover, the DAG obtained by SELF-Tree is closer to the true causal directions at the leaf nodes with higher proportion. Similar conclusions were also drawn in the subsequent discussions on indegree centrality and sample size. Each index clearly distinguishes the performance of SELF-Tree model on correct DAGs (at the diagonal of heatmap) versus incorrect ones (at the off-diagonal of heatmap). As the number of variables increases, differences in SHD, FNorm, and TPR between correct and incorrect DAGs become more pronounced, while FPR better distinguishes DAGs with fewer variables.

**Figure 7:**
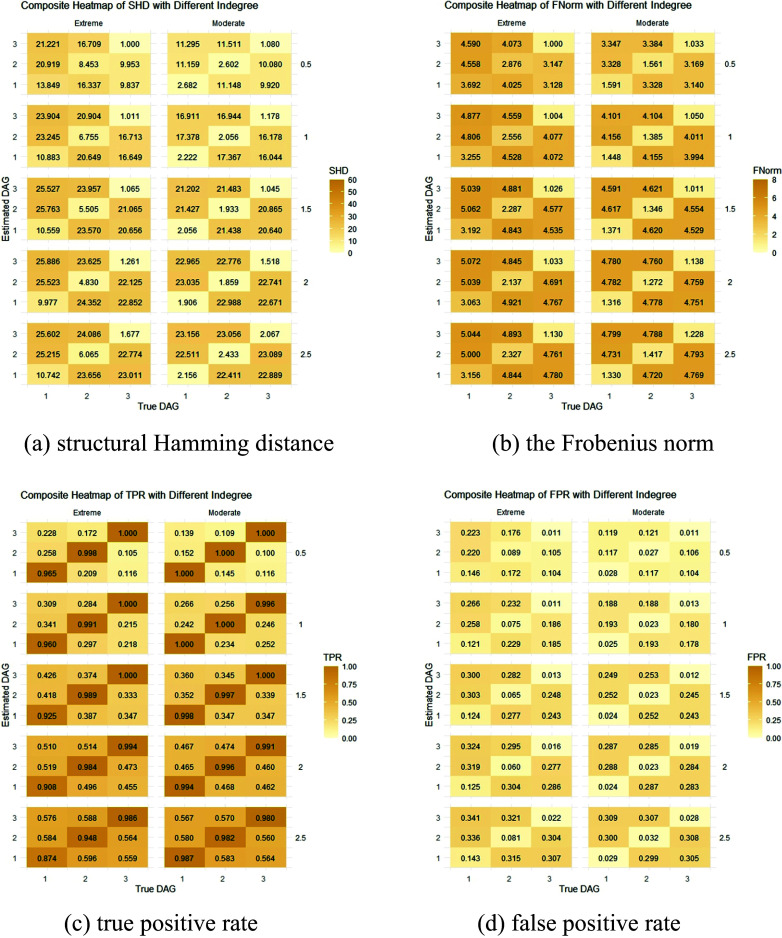
The impact of indegree centrality on the identification of heterogeneous DAGs. Figure 7 long description.

Figure 7 shows the impact of indegree centrality on heterogenous DAGs recognition when the sample size is fixed at 2,000 and the number of variables is 10. For SHD, FNorm, and FPR, correct and incorrect DAGs cannot be distinguished under *Extreme* conditions with low indegree centrality. When indegree centrality exceeds 1.0, changes in it do not significantly affect these three indicators. The TPR index can effectively distinguish correct and incorrect DAGs under all conditions, but higher indegree centrality leads to a worse performance on the incorrect DAGs identification.

**Figure 8:**
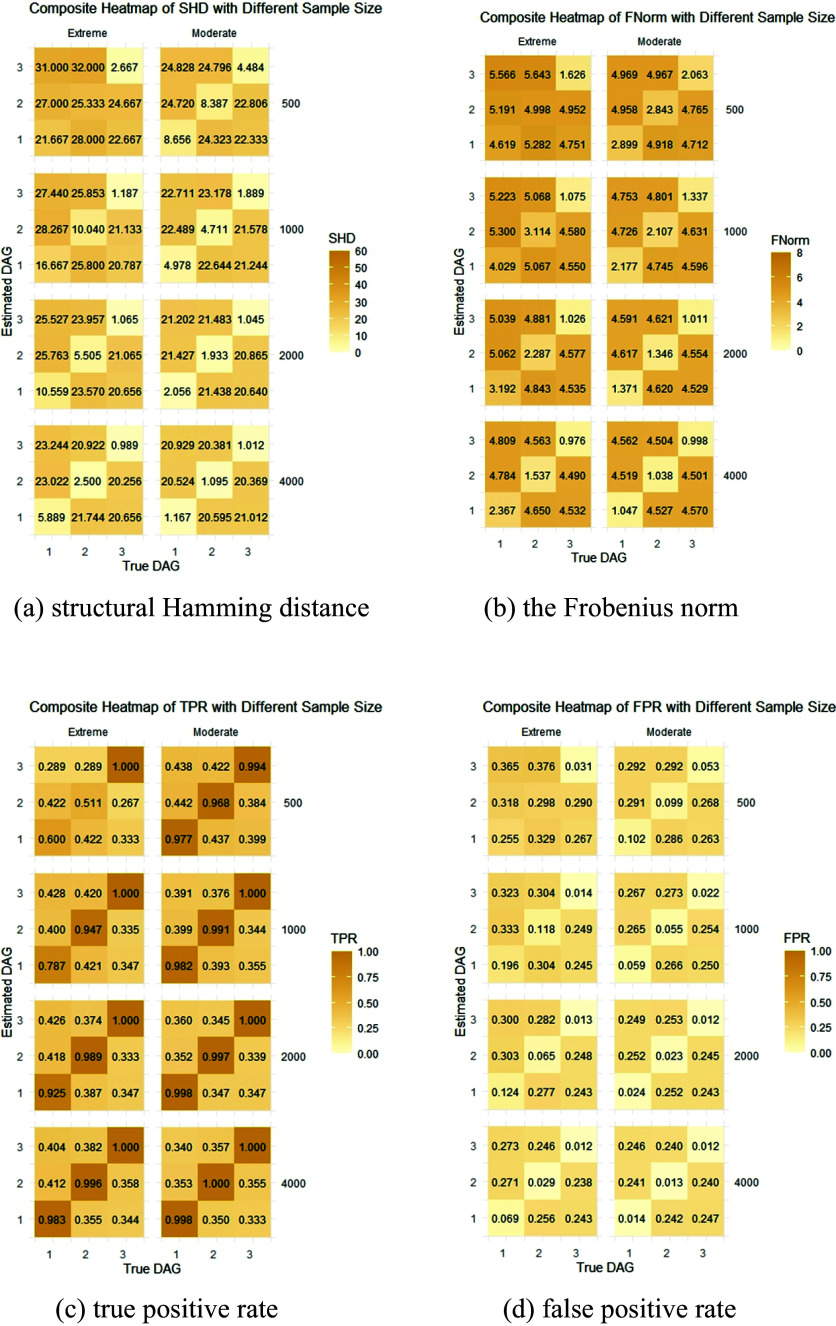
The impact of sample size on the identification of heterogeneous DAGs. Figure 8 long description.

The impact of sample size on heterogeneous DAGs identification is presented in Figure 8, with the fixed indegree centrality at 1.5 and the number of variables at 10. In *Moderate* conditions, the change of sample size does not obviously affect the values of indicators, and with a sufficiently large sample size (≥4,000), the identification of all the three heterogeneous DAGs performs similarly. On the contrary, under the *Extreme* conditions, with a sample size of 500, none of the indicators can effectively distinguish between the true DAG and the estimated DAG based on the SELF-Tree model.

In summary, the recognition of the tree structure and the identification of the heterogeneous causal directions based on SELF-Tree model are significantly influenced by the proportion of participants following different probability distributions of causal directions. The model performs better under the *Moderate* structure than the *Extreme* condition, whether the identification is focused on the tree-node, split points, and the heterogeneous causal directions.

#### 4.4.3 The identification results with spurious covariates

**Figure 9:**
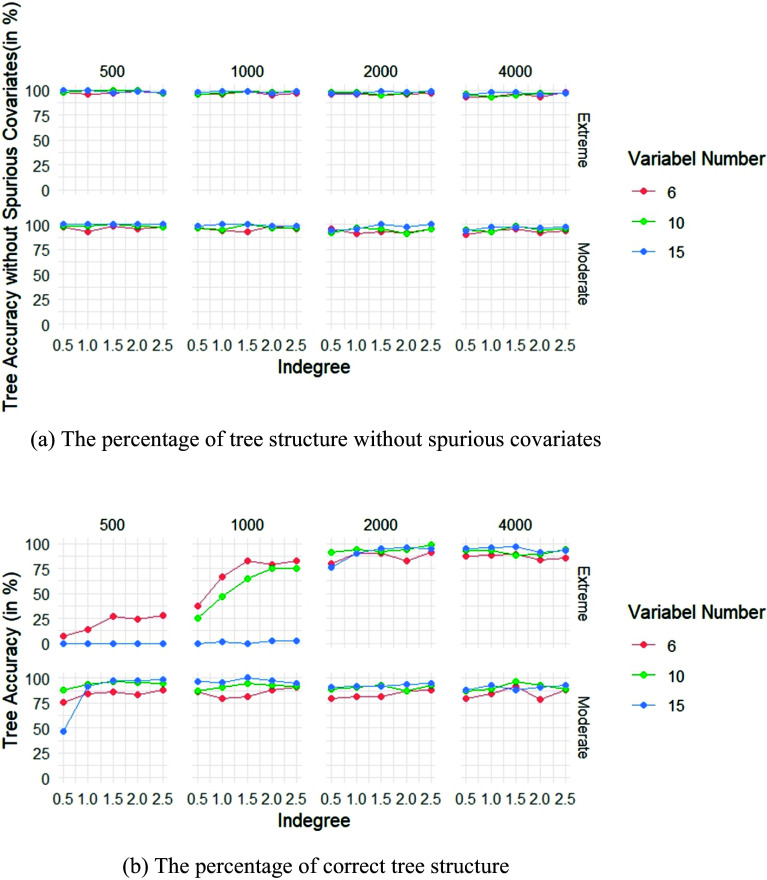
The identification result of tree structure with spurious covariates. Figure 9 long description.

This study further investigates the identification performance of SELF-Tree when covariates that are unrelated to the direction of heterogeneous causal effects are included. To this end, we repeated the experiments while adding two irrelevant covariates, $Z_{3}$ and $Z_{4}$ . As shown in Figure 9a, across 100 replications, more than 90% of the fitted SELF-Tree models did not incorporate the spurious covariates. Figure 9b illustrates the accuracy of the identified tree structures, whose performance is virtually indistinguishable from that of SELF-Tree models built without the irrelevant covariates. In other words, the inclusion of spurious covariates has little influence on the tree-structure identification of SELF-Tree.

## 5 Application

To explore the identification and explanation effect of the SELF-Tree model on the heterogeneous causal directions among multiple variables, we further conducted an empirical data analysis. Previous studies have shown that individual differences in personality traits can influence their drug consumption patterns. For instance, high levels of neuroticism and sensation-seeking have been linked to increased drug consumption (Fehrman et al., 2017). Thompson et al. (2024) used the neural network method to investigate the influence of neuroticism and sensation-seeking on the causal direction of recreational drug consumption patterns and highlighted the need for individualized risk-mitigation strategies to combat drug abuse. In their study, the distinction between low-scoring and high-scoring graphs was chosen by researchers based on the 0.1 and 0.9 quantiles. Our study leverages the data-driven approach of the SELF-Tree method to automatically identify the heterogeneous causal directions about recreational drug consumption with the change of neuroticism and sensation-seeking. We aim to provide a more objective understanding of how these personality traits influence drug consumption patterns across different subgroups of individuals.

**Figure 10:**
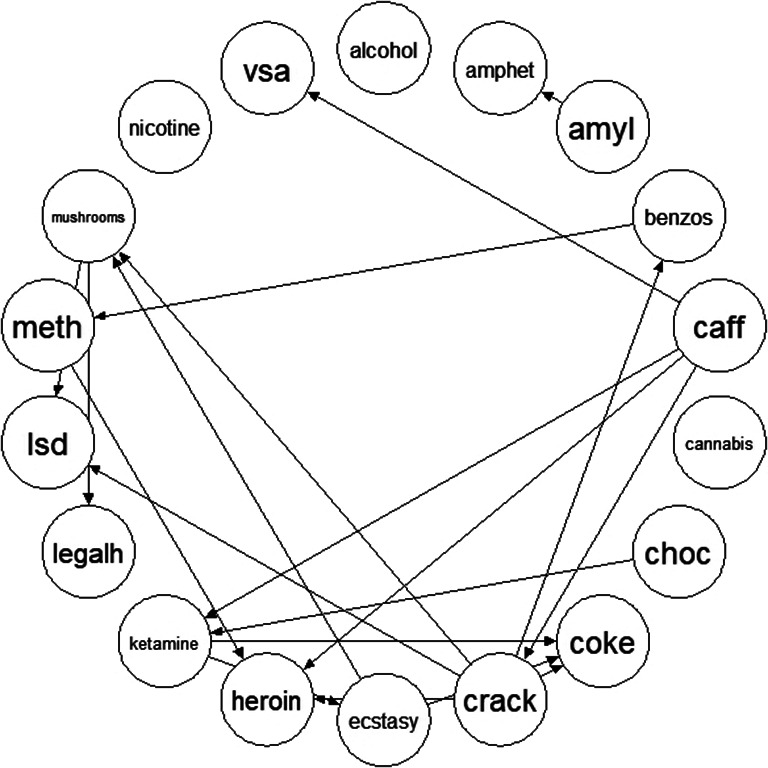
The causal direction identification of drug consumption about total participates with the following abbreviations. Amphet: amphetamine; amyl: amyl nitrite; benzos: benzodiazepine; caff: caffeine; choc: chocolate; coke: cocaine; legalh: legal high; LSD: lysergic acid diethylamide; meth: methadone; VSA: volatile substance abuse (e.g., solvents, petrol, etc.). These abbreviations apply to subsequent content as well. Figure 10 long description.

This empirical analysis employs the drug consumption dataset sourced from Fehrman et al. (2017)^4^. It records the usage of 18 drugs with seven levels^5^ by 1,885 participants. This study treats drug consumption as a continuous variable. The dataset also includes measurements of neuroticism via the NEO-FFI-R scale (McCrae & Costa, 2004) and sensation-seeking through the Impulsiveness Sensation-Seeking scale (Zuckerman, 1994). The data were collected on a 7-point Likert scale, so we treated them as continuous (Preston & Colman, 2000). We applied linear estimators and a log-likelihood function to identify the DAG structure via the SELF model A at each leaf node of CTree. We also employed the SELF model on all participant data to identify causal directions in Figure 10 (the descriptive statistics result is shown in Appendix A). The analysis used the *ucimlrepo* package in Python to read data and the *reticulate* package to connect Python and R. The analyzing code was given in Online Resource.

**Figure 11:**
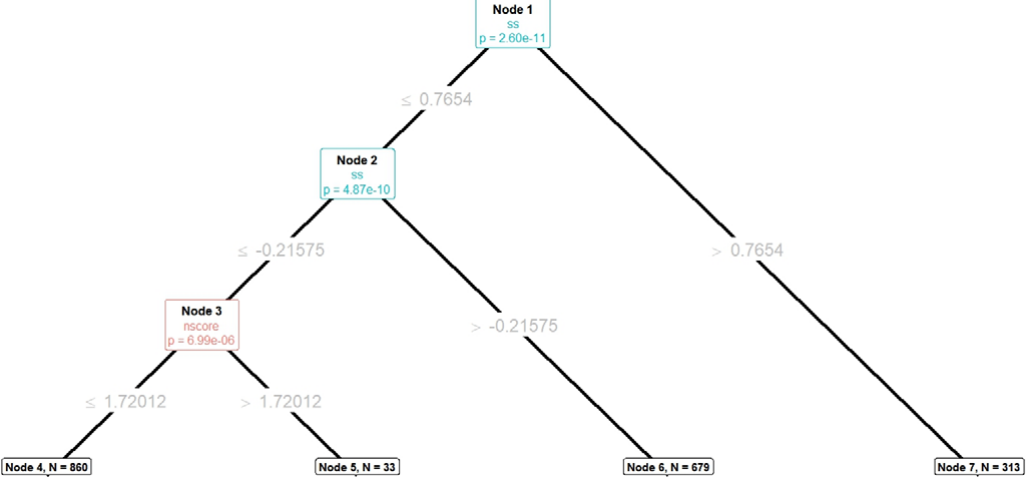
The identification result about the tree structure in drug consumption with the following abbreviations. Nscore: neuroticism; ss: sensation-seeking. Figure 11 long description.

**Figure 12:**
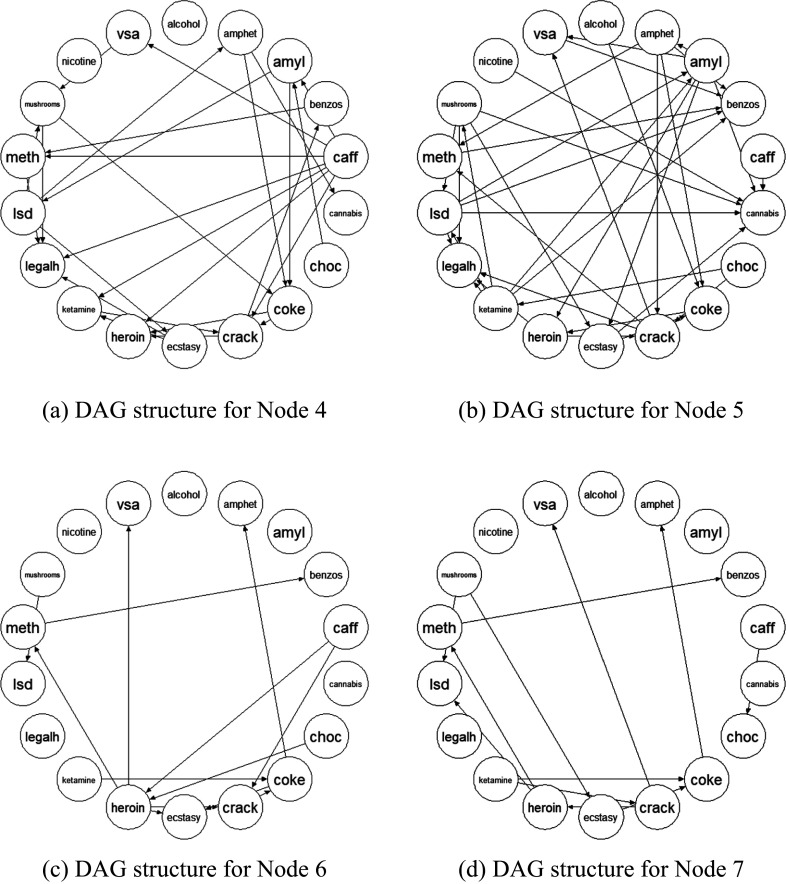
The identification result about heterogeneity in every leaf node about SELF-Tree model. Figure 12 long description.

The tree structure formed by neuroticism and sensation-seeking is shown in Figure 11, with causal directions for each leaf node in Figure 12. The first two splits in the tree model are based on sensation-seeking values, indicating its stronger impact on drug consumption causal directions. Further analysis of different DAG structures reveals that participants with high sensation-seeking have lower indegree centrality, meaning sparser variable connections. We further classified participants in each leaf node into users and nonusers based on drug use within the last decade. Descriptive statistics show that from Node 4 to Node 7 (see Appendix A for more details), the consumption of illegal drugs like amphetamine, ecstasy, heroin, and mushrooms progressively increases. This indicates that as sensation-seeking and neuroticism rise, so does the consumption level of illegal drugs.

The identification of heterogeneous DAGs at every leaf node and that of the DAGs based on the overall data are compared in Table 1. The SHD and FNorm results for different DAG structures are shown. Compared to the overall data DAG results, Nodes 4 and 5 show significant differences in the causal direction. Moreover, except for Nodes 6 and 7, which have small structural differences, the DAG structures of other leaf nodes are markedly different.

**Table 1:** The difference between heterogeneous DAGs and DAG based on the overall data Table 1 long description.

	Overall	Node 4	Node 5	Node 6	Node 7
Overall	-	30	46	22	23
Node 4	5.477	-	58	36	35
Node 5	6.782	7.616	-	44	39
Node 6	4.690	6.000	6.633	-	15
Node 7	4.796	5.916	6.245	3.873	-

*Note:* The upper triangle is the SHD matrix, and the lower triangle is the Frobenius norm. The “Overall” represents DAG in Figure 11, while “Node 4” to “Node 7” represent DAGs in Figure 12a–d), respectively.

The results of the SELF-Tree model reveal that the causal relationships in drug consumption vary significantly under different levels of neuroticism and sensation-seeking, highlighting the importance of considering heterogeneous causal directions in actual analysis. Previous research has indicated that high sensation-seekers are more susceptible to the euphoric effects of substances, thereby increasing the risk of addiction and escalating drug use (Stoops et al., 2007); this trait is also robustly associated with the consumption of harder drugs such as ecstasy (Le Bon et al., 2004; Martins et al., 2008). Specifically, the present study offers an integrated account of how this psychological disposition explains the co-occurrence of multiple forms of drug use. We found that high sensation-seeking is linked to more severe drug abuse. The sparser DAGs were observed among high sensation-seeking participants plausibly reflecting the consolidation of substance-specific habits: individuals with elevated sensation-seeking scores have already established well-defined dependency pathways in which legal or readily accessible compounds (e.g., caffeine and ketamine) function as gateway substances, whereas illicit drugs (e.g., LSD and VSA) appear as later-stage outcomes. In contrast, low sensation-seekers have yet to crystallize any comparable dependency structure in the DAGs; consequently, their substance-use patterns remain diffuse, with multiple unordered transitions emanating from each drug and no dominant directional sequence. The SELF-Tree model not only enhances the accuracy of identifying heterogeneous causal directions but also offers valuable insights for developing targeted interventions to address drug abuse.

## 6 Discussion

### 6.1 Research implications

The present investigation is devoted to developing an interpretable framework for the exploratory identification of heterogeneous causal directions among multiple psychological variables. Leveraging insights from psychometrics, structural equation modeling, and the burgeoning literature on causal inference and discovery, we instantiate interpretability via recursive partitioning. Capitalizing on the SELF framework’s capacity to integrate global- and local-view perspectives (Cai et al., 2018), we deploy it as a vehicle for directional causal identification. Nevertheless, our methodological architecture is model-agnostic; subsequent researchers may readily substitute alternative causal-discovery engines while retaining the recursive-partitioning backbone.

More fundamentally, this work confronts a perennial yet under-examined conundrum in psychological science: adjudicating “chicken-or-egg” causal priority when experimental manipulation is impracticable. We contend that participant heterogeneity furnishes a principled analytic lens. Although the dominant paradigm assumes population homogeneity, a venerable tradition has sought to formalize heterogeneity—beginning with differential covariance structures, proceeding to heterogeneous treatment-effect models (Athey & Imbens, 2016; Hill, 2011), and culminating in recent treatments of heterogeneous causal directions between two variables (Wiedermann et al., 2024). Such heterogeneity is posited to arise from stable individual differences or transient contextual factors, operationalized empirically as covariates. We extend this lineage by modeling multivariate causal-direction heterogeneity, thereby addressing the chicken-or-egg question: causal precedence may shift as a function of environmental affordances, personality configurations, or even ephemeral situational perturbations captured at the moment of assessment.

Our analytic strategy presumes non-Gaussian data—a distributional stance that may appear unconventional yet is empirically ubiquitous. Contemporary test scores (Ho & Yu, 2015) and psychopathological traits (Rosenström et al., 2023) routinely exhibit marked departures from normality, and recent psychometric scholarship has explicitly theorized heterogeneity within non-Gaussian distributions (e.g., Asparouhov & Muthén, 2016; Son et al., 2019). Consequently, our modeling choices remain firmly within the disciplinary mainstream.

When the variable space is high-dimensional, the visualization and substantive interpretation of the resultant directed acyclic graphs (DAGs) become nontrivial. We therefore propose a bifurcated representational scheme: the recursive tree structure is displayed separately from the DAGs embedded within each terminal node. Regarding causal interpretation, two complementary layers are required. First, the requisite identifying assumptions—most notably causal sufficiency and the distributional characteristics of the data—must be transparently stipulated; under these premises, directed edges may be endowed with causal meaning. Second, in the presence of directional heterogeneity, we advocate supplementing each DAG with descriptive statistics of the constituent variables and applying graph-theoretic diagnostics—e.g., centrality indices and community-detection algorithms—to explicate cross-contextual differences in causal architectures.

### 6.2 Limitation and future directions

Data-driven causal-direction identification is by no means infallible; its validity hinges on a constellation of assumptions and stringent data requirements. Among these, causal sufficiency—i.e., the premise that the measured set includes every variable that participates in the underlying causal graph (Cai et al., 2018; Glymour et al., 2019; Zhou et al., 2023)—is pivotal. Unmeasured common causes can induce spurious associations, thereby distorting the recovered causal directions (Richardson & Spirtes, 2002). The current SELF-Tree implementation presupposes causal sufficiency, and the SELF model at its core is not equipped to accommodate latent confounders. A straightforward remedy is to replace SELF with algorithms that explicitly model hidden variables; however, each alternative imposes its own auxiliary restrictions. For instance, Frot et al. (2019) require a single latent factor that linearly influences all observed variables. Meanwhile, whether CTree procedures can detect heterogeneous associations when causal sufficiency is violated remains an open empirical question that may necessitate substantial refinements to recursive partitioning.

As is the case with hidden common causes, the possible omission of influential covariates constitutes an open issue that deserves closer scrutiny. A key premise underlying the SELF-Tree framework is that every covariate capable of generating heterogeneity must be supplied to the recursive-partitioning engine (Assumption 2). This requirement is stringent: in practice, it is seldom possible to guarantee that all relevant attributes have been measured. Consequently, most studies that target directionally heterogeneous effects either tacitly assume covariate sufficiency (e.g., Ni et al., 2019; Thompson et al., 2024) or ignore the issue altogether (e.g., Zhou et al., 2023). Recent work nevertheless demonstrates that omitting important moderators can render heterogeneous causal directions undetectable, leading to the erroneous conclusion that the causal relation is homogeneous across the sample (Fokkema et al., 2025; Wiedermann et al., 2024). We therefore argue that the adequacy of the covariate set remains an active frontier that future research must continue to explore.

Extensive recursive-partitioning studies have shown that the algorithm continues to single out the truly informative covariates even when a large pool of noise variables is added to the model (e.g., Brandmaier et al., 2013; Fokkema & Zeileis, 2024; Jones et al., 2020; Wiedermann et al., 2024). Our simulation evidence also indicates that spurious covariates exert only negligible influence on identification accuracy. Nevertheless, such a method provides only an indirect safeguard against the fundamental problem of covariate sufficiency. Future work should therefore explore data-driven screening procedures that actively select the most relevant covariates before invoking the SELF-Tree routine. Actually, unlike structural equation modeling or generalized linear mixed models, it does not presuppose a pre-specified functional form for variable relations in SELF-Tree model. Consequently, given a sufficiently rich feature space, one could, in principle, automate the discovery of covariates that drive directional heterogeneity and subsequently construct the SELF-Tree. Such an endeavor presupposes, of course, that the available measurements exhaustively capture the relevant covariate space.

Beyond the core assumptions, we impose several commonly adopted constraints to safeguard the validity of SELF-Tree, such as variables and covariates are free of measurement error (Gische & Voelkle, 2022), and covariates are mutually independent (Ni et al., 2019; Thompson et al., 2024). Although routinely invoked, these idealizations can materially distort identification. Self-reported drug-use data, for instance, are vulnerable to social-desirability bias (Bispo Júnior, 2022; Larson, 2019), inducing distributional shapes that reflect measurement artefacts rather than true scores. Such contamination can propagate into the causal graph, and—contrary to classical attenuation—the divergence from the true structure may intensify with sample size (Wiedermann et al., 2018; Yang et al., 2022; Zhang et al., 2018). Likewise, emerging work shows that recursive-partitioning performance degrades when covariates are collinear or stochastically dependent (Fokkema et al., 2018; Fokkema & Zeileis, 2024; Wiedermann et al., 2024). Whether SELF-Tree remains robust under realistic response biases and dependency patterns is therefore an open empirical question warrants systematic investigation.

The recursive partitioning method offers exceptional interpretability, yet it is accompanied by well-documented limitations: sensitivity to minor data perturbations, proneness to overfitting, and convergence to locally optimal solutions. Ensemble-learning strategies that aggregate multiple tree-based learners into a more robust predictive mechanism—commonly instantiated as random forests (Strobl et al., 2009)—provide a compelling antidote. Brandmaier et al. (2016) have already translated this insight into the structural equation modelling forest. We contend that an analogous ensemble extension of SELF-Tree would substantially enhance the reliability of multivariate heterogeneous causal direction discovery.

In sum, integrating recursive partitioning into the broader causal-discovery toolkit to address heterogeneity in multivariate causal directions remains a fertile research frontier. Continued methodological refinements to recursive partitioning itself are likely to constitute a central focus for subsequent inquiry.

## 7 Conclusion

This study introduces the SELF-Tree model to identify and explain heterogeneous causal directions among multiple variables. As a two-step model, it constructs a covariate tree non-parametrically and uses a structural equation likelihood framework at each leaf node to determine causal directions. Simulation studies show the model effectively recognizes heterogeneous DAGs under *Moderate* structures. Empirical analysis on drug consumption data further demonstrates its ability to reveal causal relationship heterogeneity among individuals with different sensation-seeking levels. In summary, this study develops an explanatory modeling method for multivariable causal relationships using tree models and causal-discovery techniques. It highlights the significance of tree models in detecting heterogeneous causal directions and offers an efficient toolkit for future psychological research.

## Supporting information

10.1017/psy.2025.10067.sm001 Li and Wen supplementary material Li and Wen supplementary material

## Figures and Tables

**Figure A1:**
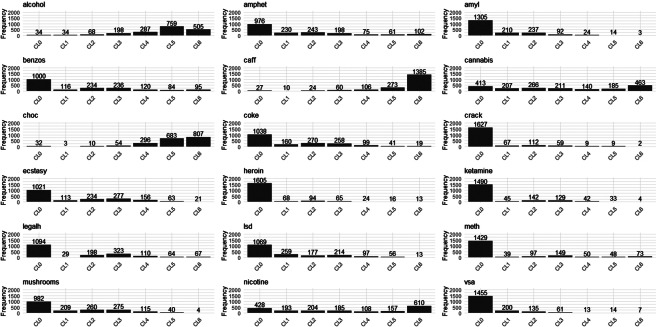
The description statistics on the total samples. Figure A1 long description.

**Figure A2:**
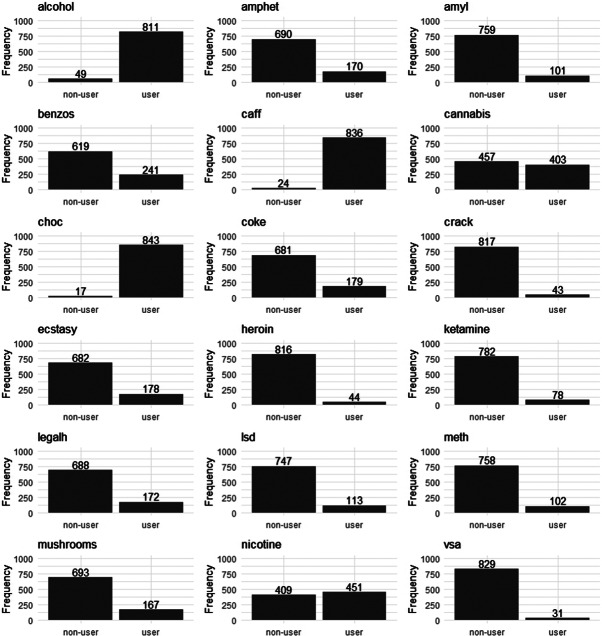
The description statistics on Node 4 about the heterogeneous drug consumption patterns. Note that the “user” represents the participants who used the drug in the last 10 years, while “non-user” represents the participants who never used the drug or used it over a decade. This definition applies to subsequent mentions of user and nonuser. Figure A2 long description.

**Figure A3:**
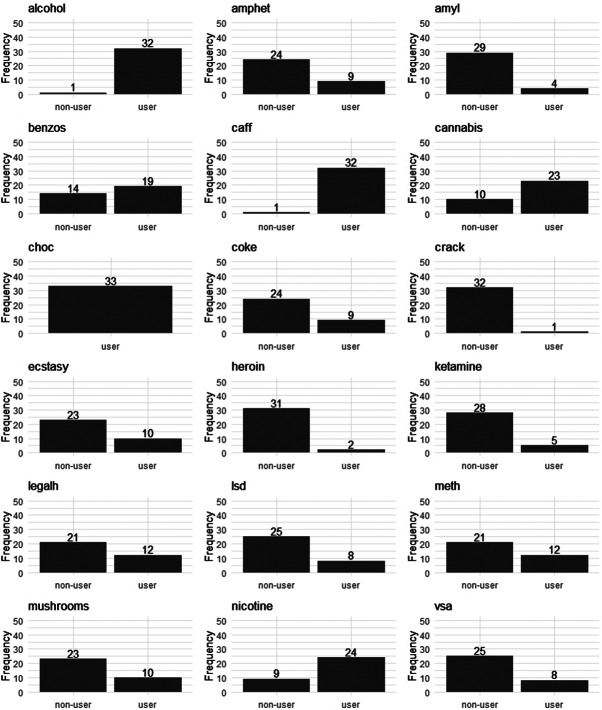
The description statistics on Node 5 about the heterogeneous drug consumption patterns. Figure A3 long description.

**Figure A4:**
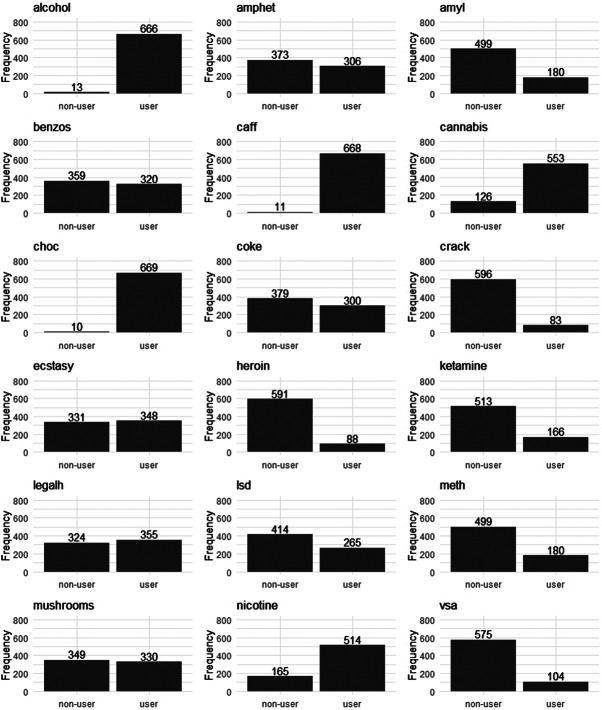
The description statistics on Node 6 about the heterogeneous drug consumption patterns. Figure A4 long description.

**Figure A5:**
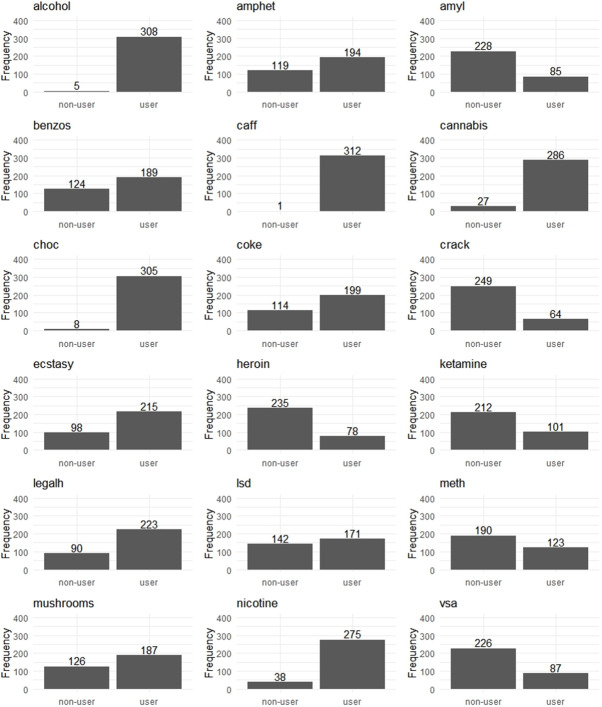
The description statistics on Node 7 about the heterogeneous drug consumption patterns. Figure A5 long description.

## Data Availability

We have shared the data and code in the supplement materials.

## Footnotes

1 As formula (1), the probability factorization of $X_{i}\leftarrow X_{j}\to X_{k}$ is $\mathrm{Pr}\left(X_{i},X_{j},X_{k}\right)=\mathrm{Pr}\left(X_{j}\right)\times \mathrm{Pr}\left(X_{i}\right|X_{j})\times \mathrm{Pr}\left(X_{k}\right|X_{j})$ , while in $X_{i}\to X_{j}\to X_{k}$ condition, there is $\mathrm{Pr}\left(X_{i},X_{j},X_{k}\right)=\mathrm{Pr}\left(X_{i}\right)\times \mathrm{Pr}\left(X_{j}\right|X_{i})\times \mathrm{Pr}\left(X_{k}\right|X_{j})=\mathrm{Pr}\left(X_{j}\right)\times \mathrm{Pr}\left(X_{i}\right|X_{j})\times \mathrm{Pr}\left(X_{k}\right|X_{j})$ , with the conditional probability $\mathrm{Pr}\left(X_{i}\right)\times \mathrm{Pr}\left(X_{j}\right|X_{i})=\mathrm{Pr}\left(X_{j}\right)\times \mathrm{Pr}\left(X_{i}\right|X_{j})$ .

2 We use *tree-node* to differentiate the “node” term commonly used in both DAG and tree models.

3 The synthetic data were generated with the *synthetic_data_linear()* function embedded in the *SELF* package (Cai et al., 2018); the heavy-tailed and heteroskedastic noise characteristics are automatically induced by the default parameter settings of this routine.

4 The data was retrieved from *the UCI Machine Learning Repository* https://archive.ics.uci.edu

5 The levels are labeled as follows. CL0: Never Used, CL1: Used Over a Decade Ago, CL2: Used in Last Decade, CL3: Used in Last Year, CL4: Used in Last Month, CL5: Used in Last Week and CL6: Used in Last Day.

## Supplementary material

The supplementary material for this article can be found at https://doi.org/10.1017/psy.2025.10067.

## AI usage

During the preparation of this manuscript, the authors used Kimi (publicly available at https://kimi.moonshot.cn, developed by Moonshot AI Co., Ltd.) exclusively to enhance the readability and language of the submitted manuscript. No proprietary data were uploaded, and no modifications were made to the tool’s public corpus. The Kimi was employed on March 18, 2025 (the original submission), August 10, 2025 (revision after the first round of peer review), and November 18, 2025 (final revision after second round of peer review) in order to improve the readability and language of the manuscript. After each session, the authors reviewed, edited, and approved every change, and we accept full responsibility for the final manuscript. The use of this AI tool does not introduce any competing interests or potential bias.

## Appendix A

Appendix A provides the descriptive statistics results in the application of the SELF-Tree model on heterogeneous drug consumption pattern identification.

## Figure 2 Long description

At the top center is node Z, which branches left for Z less than or equal to zero and right for Z greater than zero. The left branch leads to X sub 1, which splits to X sub 2 and X sub 4. X sub 2 connects downward to X sub 3, then to X sub 5. The right branch leads to X sub 2, which splits to X sub 1 and X sub 3. X sub 1 connects downward to X sub 5. X sub 4 is isolated on the right side.

Navigate back to Figure 2.

## Figure 3 Long description

The left panel shows the moderate structure. At the top is node Z 1, which branches to Z 2 and D A G 3. The left branch from Z 1 to Z 2 is labeled less than or equal to zero, and the right branch to D A G 3 is labeled greater than zero, with D A G 3 containing 50 percent. Z 2 further branches left to D A G 1, labeled less than or equal to zero, and right to D A G 2, labeled greater than zero. D A G 1 and D A G 2 each contain 25 percent. The right panel shows the extreme structure. At the top is node Z 1, which branches to Z 2 and D A G 3. The left branch from Z 1 to Z 2 is labeled less than or equal to negative one, and the right branch to D A G 3 is labeled greater than negative one, with D A G 3 containing 84.0 percent. Z 2 further branches left to D A G 1, labeled less than or equal to negative one, and right to D A G 2, labeled greater than negative one. D A G 1 contains 5.3 percent and D A G 2 contains 10.7 percent.

Navigate back to Figure 3.

## Figure 4 Long description

Panels are organized with sample sizes increasing from left (500) to right (4000) and conditions labeled as Extreme (top row) and Moderate (bottom row). Each panel plots tree accuracy (y-axis, 0 to 100 percent) against indegree (x-axis, 0.5 to 2.5). Three lines per panel: red for variable number 6, green for 10, blue for 15. In the Extreme condition, tree accuracy for variable number 6 increases with indegree and sample size, while variable numbers 10 and 15 remain low and flat. In the Moderate condition, all variable numbers show high accuracy, with variable number 6 starting lower at small sample size but converging with others as sample size increases. Legend at right identifies line colors. Gridlines present in all panels.

Navigate back to Figure 4.

## Figure 5 Long description

The layout consists of two main panels. The top panel, labeled Moderate condition, contains eight line graphs in a 2 by 4 grid. The x-axis of each graph is labeled Indegree, ranging from 0.5 to 2.5. The y-axis is labeled Split Accuracy for Moderate Structure (Bias), ranging from 0 to 0.2. Columns represent sample sizes 500, 1000, 2000, and 4000, increasing left to right. Rows represent Z1 (top) and Z2 (bottom). Each graph contains three lines: red for variable number 6, green for 10, and blue for 15. For Z1, all lines are nearly flat and close to zero across all sample sizes. For Z2, bias is highest at low indegree and decreases as indegree increases, with the effect diminishing as sample size increases. The bottom panel, labeled Extreme condition, follows the same structure. The y-axis is labeled Split Accuracy for Extreme Structure (Bias), ranging from 0 to 0.6. For Z1, only the red line (variable number 6) is present for sample size 500, and all lines are absent for higher variable numbers and sample sizes. For Z2, bias is highest at low sample size and low indegree, especially for variable number 6, and decreases as sample size and indegree increase. The legend at the right identifies line colors for variable numbers 6, 10, and 15. The caption notes that some results are omitted for the Extreme condition at higher variable numbers and lower sample sizes.

Navigate back to Figure 5.

## Figure 6 Long description

Starting at the top-left, panel a shows structural Hamming distance with columns labeled 1, 2, 3 for true D A Gs and rows for estimated D A Gs, subdivided by variable numbers 6, 10, 15 and scenarios Extreme, Moderate. Color intensity increases with higher values, ranging from 0.869 to 50.131. Panel b, top-right, displays Frobenius norm with similar axes and subdivisions, values from 0.854 to 7.071. Panel c, bottom-left, presents true positive rate, values from 0.218 to 1.000, with darker cells along the diagonal. Panel d, bottom-right, shows false positive rate, values from 0.005 to 0.300, with lighter diagonal cells. Each heatmap uses a color scale bar for quantitative mapping. Greater diagonal contrast indicates improved identification of heterogeneous causal directions.

Navigate back to Figure 6.

## Figure 7 Long description

Top-left panel shows structural Hamming distance with estimated D A G on the y-axis and true D A G on the x-axis, values ranging from 1.000 to 40.640, with higher values in the bottom rows and right columns. Top-right panel displays Frobenius norm, values from 1.000 to 6.140, with higher values concentrated in the lower right. Bottom-left panel presents true positive rate, values from 0.000 to 1.000, with highest rates along the diagonal and upper rows. Bottom-right panel shows false positive rate, values from 0.011 to 0.307, with lowest rates in the upper left and highest in the lower right. Each panel is split into Extreme and Moderate indegree columns. Color intensity increases with metric value. All axes are labeled from 1 to 3.

Navigate back to Figure 7.

## Figure 8 Long description

Starting at the top-left, the structural Hamming distance heatmap displays estimated D A G versus true D A G for sample sizes 500, 1000, 2000, and 4000, with values ranging from 1.012 to 31.000. The extreme and moderate columns show decreasing S H D as sample size increases. The top-right panel shows Frobenius norm values from 0.263 to 5.643, also decreasing with larger sample sizes. The bottom-left panel presents true positive rate, ranging from 0.267 to 1.000, with higher values for moderate conditions and larger samples. The bottom-right panel shows false positive rate, ranging from 0.012 to 0.376, with lower values for moderate conditions and larger samples. Each heatmap includes a color scale bar and axes labeled estimated D A G and true D A G.

Navigate back to Figure 8.

## Figure 9 Long description

The layout consists of two main blocks, each with four columns and two rows. The x-axis in all panels is Indegree, ranging from 0.5 to 2.5. The y-axis in the top block is labeled Tree Accuracy without Spurious Covariates (in percent), and in the bottom block Tree Accuracy (in percent), both ranging from 0 to 100. Columns are labeled by sample size: 500, 1000, 2000, 4000. Rows are labeled by condition: Extreme (top), Moderate (bottom). Each panel contains three lines: red for variable number 6, green for 10, blue for 15. In the top block, all lines remain near 100 percent across all indegree and sample sizes, indicating consistently high accuracy without spurious covariates. In the bottom block, accuracy increases with sample size and indegree, especially for variable numbers 10 and 15, with the most pronounced improvement between sample sizes 500 and 1000. The legend on the right identifies line colors by variable number.

Navigate back to Figure 9.

## Figure 10 Long description

Starting at the top center, V S A is linked by arrows from caff and meth. Alcohol, amphet, amyl, benzos, caff, cannabis, choc, coke, crack, ecstasy, heroin, ketamine, legalh, L S D, meth, mushrooms, and nicotine are arranged clockwise. Caff points to benzos, choc, coke, crack, ecstasy, heroin, ketamine, legalh, L S D, meth, mushrooms, nicotine, and V S A. Meth points to mushrooms, L S D, legalh, heroin, crack, and V S A. Crack receives arrows from coke, heroin, and ecstasy. Heroin receives arrows from ketamine, legalh, L S D, meth, mushrooms, and caff. Ecstasy receives arrows from crack and heroin. Legalh receives arrows from L S D and meth. Mushrooms receive arrows from meth and L S D. Benzo receives an arrow from caff. V S A receives arrows from meth and caff. All arrows indicate the direction of causal influence between drug types.

Navigate back to Figure 10.

## Figure 11 Long description

Starting at the top, Node 1 uses sensation-seeking (ss) with p equals 2 point 60 e minus 11. The left branch splits at Node 2, also ss, p equals 4 point 87 e minus 10, with threshold less than or equal to 0 point 7654. Node 2’s left branch leads to Node 3, neuroticism score (nscore), p equals 6 point 99 e minus 6, with threshold less than or equal to minus 0 point 21575. Node 3 splits into Node 4, N equals 860 for nscore less than or equal to 1 point 72012, and Node 5, N equals 33 for nscore greater than 1 point 72012. Node 2’s right branch leads to Node 6, N equals 679 for ss greater than minus 0 point 21575. Node 1’s right branch leads to Node 7, N equals 313 for ss greater than 0 point 7654. All terminal nodes display sample counts (N).

Navigate back to Figure 11.

## Figure 12 Long description

Panel a at top-left shows the D A G structure for Node 4 with nodes labeled meth, l s d, legalh, ketamine, heroin, ecstasy, crack, coke, choc, cannabis, caff, benzos, amyl, amphet, alcohol, v s a, nicotine, and mushrooms. Directed edges connect meth to multiple nodes including l s d, legalh, heroin, crack, and coke. Amyl, caff, choc, and coke are highly connected. Panel b at top-right shows Node 5 with similar node labels but more dense connections, especially from meth, caff, and crack to other nodes. Panel c at bottom-left for Node 6 shows fewer connections, with meth, caff, and crack as main hubs. Panel d at bottom-right for Node 7 shows even sparser connections, with meth, caff, and crack still central but with fewer outgoing edges. All panels use the same set of node labels and circular arrangement, but the pattern and density of directed edges differ, illustrating heterogeneity in D A G structures across nodes.

Navigate back to Figure 12.

## Table 1 Long description

The table consists of five columns and five rows labeled Overall, Node 4, Node 5, Node 6, and Node 7. The upper triangle contains S H D values, and the lower triangle contains Frobenius norm values. The diagonal cells are marked with dashes. From left to right, the first row labeled Overall has values: dash, 30, 46, 22, 23. The second row, Node 4, has 5.477, dash, 58, 36, 35. The third row, Node 5, has 6.782, 7.616, dash, 44, 39. The fourth row, Node 6, has 4.690, 6.000, 6.633, dash, 15. The fifth row, Node 7, has 4.796, 5.916, 6.245, 3.873, dash. The upper triangle values represent S H D matrix entries, and the lower triangle values represent Frobenius norms. The Overall column and row refer to the D A G in Figure 11, while Node 4 to Node 7 refer to D A Gs in Figure 12 panels a through d.

Navigate back to Table 1.

## Figure A1 Long description

From the top-left, each panel displays a bar chart for a specific substance. The x-axis is labeled CL0 to CL6, and the y-axis is labeled Frequency. Alcohol: CL0 34, CL1 34, CL2 68, CL3 198, CL4 287, CL5 759, CL6 505. Amphet: CL0 976, CL1 230, CL2 243, CL3 198, CL4 75, CL5 61, CL6 102. Amyl: CL0 1305, CL1 210, CL2 237, CL3 92, CL4 24, CL5 14, CL6 3. Benzos: CL0 1000, CL1 116, CL2 234, CL3 236, CL4 120, CL5 84, CL6 95. Caff: CL0 27, CL1 10, CL2 24, CL3 60, CL4 106, CL5 273, CL6 1385. Cannabis: CL0 413, CL1 207, CL2 266, CL3 211, CL4 140, CL5 185, CL6 463. Choc: CL0 32, CL1 3, CL2 10, CL3 54, CL4 296, CL5 683, CL6 807. Coke: CL0 1038, CL1 160, CL2 270, CL3 258, CL4 99, CL5 41, CL6 19. Crack: CL0 1627, CL1 67, CL2 112, CL3 59, CL4 9, CL5 9, CL6 2. Ecstasy: CL0 1021, CL1 113, CL2 234, CL3 277, CL4 156, CL5 63, CL6 21. Heroin: CL0 1605, CL1 68, CL2 94, CL3 65, CL4 24, CL5 16, CL6 13. Ketamine: CL0 1490, CL1 45, CL2 142, CL3 129, CL4 42, CL5 33, CL6 4. Legalh: CL0 1094, CL1 29, CL2 198, CL3 323, CL4 110, CL5 64, CL6 67. L S D: CL0 1069, CL1 259, CL2 177, CL3 214, CL4 97, CL5 56, CL6 13. Meth: CL0 1429, CL1 39, CL2 97, CL3 149, CL4 50, CL5 48, CL6 73. Mushrooms: CL0 982, CL1 209, CL2 260, CL3 275, CL4 115, CL5 40, CL6 4. Nicotine: CL0 428, CL1 193, CL2 204, CL3 185, CL4 108, CL5 157, CL6 610. V S A: CL0 1455, CL1 200, CL2 135, CL3 61, CL4 13, CL5 14, CL6 7. Most substances show highest frequencies at CL0, except choc, caff, nicotine, and alcohol, which peak at CL5 or CL6.

Navigate back to Figure A1.

## Figure A2 Long description

Starting from the top-left and moving right, the first row displays alcohol (non-user 49, user 811), amphet (non-user 690, user 170), amyl (non-user 759, user 101), benzos (non-user 619, user 241). The second row shows caff (non-user 24, user 836), cannabis (non-user 457, user 403), choc (non-user 17, user 843), coke (non-user 681, user 179). The third row presents crack (non-user 817, user 43), ecstasy (non-user 682, user 178), heroin (non-user 816, user 44), ketamine (non-user 782, user 78). The fourth row includes legalh (non-user 688, user 172), l s d (non-user 747, user 113), meth (non-user 758, user 102), mushrooms (non-user 693, user 167). The fifth row contains nicotine (non-user 409, user 451), v s a (non-user 829, user 31). Most drugs show higher non-user frequencies, except alcohol, caff, choc, and nicotine, where user counts are comparable or higher. Each chart uses frequency as the y-axis and non-user/user as the x-axis categories.

Navigate back to Figure A2.

## Figure A3 Long description

Starting from the top-left and moving right, each panel displays a drug name above two bars labeled non-user and user. Alcohol shows 1 non-user and 32 users. Amphet has 24 non-users and 9 users. Amyl records 29 non-users and 4 users. Benzos has 14 non-users and 19 users. Caff shows 1 non-user and 32 users. Cannabis has 10 non-users and 23 users. Choc displays only 33 users. Coke has 24 non-users and 9 users. Crack shows 32 non-users and 1 user. Ecstasy has 23 non-users and 10 users. Heroin records 31 non-users and 2 users. Ketamine has 28 non-users and 5 users. Legalh shows 21 non-users and 12 users. L S D has 25 non-users and 8 users. Meth records 21 non-users and 12 users. Mushrooms has 23 non-users and 10 users. Nicotine shows 9 non-users and 24 users. V S A has 25 non-users and 8 users. Each chart uses frequency as the y-axis, with values ranging from 0 to 50. The distribution highlights that some drugs, like chocolate and caffeine, have only user bars, while others, such as crack and heroin, have predominantly non-user bars.

Navigate back to Figure A3.

## Figure A4 Long description

Starting from the top-left and moving right, each panel displays two bars labeled non-user and user, with frequency values. Alcohol shows 13 non-users and 666 users. Amphet has 373 non-users and 306 users. Amyl records 499 non-users and 180 users. Benzos show 359 non-users and 320 users. Caff has 11 non-users and 668 users. Cannabis displays 126 non-users and 553 users. Choc records 10 non-users and 669 users. Coke shows 379 non-users and 300 users. Crack has 596 non-users and 83 users. Ecstasy records 331 non-users and 348 users. Heroin shows 591 non-users and 88 users. Ketamine has 513 non-users and 166 users. Legalh records 324 non-users and 355 users. L S D shows 414 non-users and 265 users. Meth has 499 non-users and 180 users. Mushrooms record 349 non-users and 330 users. Nicotine shows 165 non-users and 514 users. V S A has 575 non-users and 104 users. The panels reveal that substances like alcohol, caffeine, chocolate, cannabis, nicotine, and legal highs have higher user frequencies, while heroin, crack, ketamine, and V S A have higher non-user frequencies.

Navigate back to Figure A4.

## Figure A5 Long description

Starting from the top-left and moving right, the first row shows alcohol with 5 non-users and 308 users, amphet with 119 non-users and 194 users, amyl with 228 non-users and 85 users, benzos with 124 non-users and 189 users. The second row displays caff with 1 non-user and 312 users, cannabis with 27 non-users and 286 users, choc with 8 non-users and 305 users, coke with 114 non-users and 199 users. The third row presents crack with 249 non-users and 64 users, ecstasy with 98 non-users and 215 users, heroin with 235 non-users and 78 users, ketamine with 212 non-users and 101 users. The fourth row includes legalh with 90 non-users and 223 users, l s d with 142 non-users and 171 users, meth with 190 non-users and 123 users, mushrooms with 126 non-users and 187 users. The final chart shows nicotine with 38 non-users and 275 users, v s a with 226 non-users and 87 users. Most drugs have higher user frequencies except amyl, crack, heroin, ketamine, meth, and v s a, where non-user counts are higher.

Navigate back to Figure A5.
