# Supplementary material for: SELF-Tree: An Interpretable Model for Multivariate Causal Direction Heterogeneity Analysis
Source: Psychometrika. 2025 Dec 10;91(2):556–86. doi: 10.1017/psy.2025.10067 (PMC13294625; doi:10.1017/psy.2025.10067)
Supplement: Li and Wen supplementary material [file S0033312325100677sup001.docx]

### ----------------------------------------------------------------------------------------- ###

### File: Real Data Analysis.R

### Purpose: This file provides the source code of SELF-Tree model application

### about evaluating the heterogeneous drug consumption patterns

### with two covariates —— neuroticism and sensation-seeking based on

### the dataset of Fehrman et al. (2017).

### Author: Zhifei Li

### E-mail: lizhifei@mail.bnu.edu.cn

### Last Mod.: March 26, 2025

### ----------------------------------------------------------------------------------------- ###

### Load necessary R packages

library(SELF)

library(partykit)

library(reticulate)

library(dplyr)

library(ggparty)

library(networktree)

library(ggplot2)

library(gridExtra)

### Load custom functions

treeinput <- function (vars, prefix = "var")

{

if (!inherits(vars, "data.frame") && !inherits(vars, "matrix")) {

vars <- data.frame(unlist(vars))

colnames(vars) <- prefix

}

if (any(is.null(colnames(vars)))) {

colnames(vars) <- paste(prefix, 1:ncol(vars), sep = "")

}

vars <- as.data.frame(vars)

return(vars)

}

SELFCTree <- function (nodevars, splitvars, model = "correlation",

transform = c("cor", "pcor", "glasso"), na.action = na.omit,

weights = NULL, ...)

{

nodevars <- treeinput(nodevars, prefix = "nodevar")

splitvars <- treeinput(splitvars, prefix = "splitvar")

netdata <- as.data.frame(nodevars)

splitvars <- as.data.frame(splitvars)

d <- cbind(splitvars, netdata)

f1 <- Formula::as.Formula(paste(c(

paste(colnames(netdata),

collapse = " + "),

" ~ ",

paste(colnames(splitvars),

collapse = " + ")

), collapse = ""))

tree <-

partykit::ctree(

formula = f1,

data = d,

ytrafo = function(data,

weights, control) {

cortrafo(data = data, weights = weights, control = NULL,

model = model)

},

na.action = na.action,

control = partykit::ctree_control(...)

)

res <- tree

return(res)

}

StructuralHammingDistance <- function(G_True, G_Hat) {

SHD <- sum(abs(G_True - G_Hat))

return(SHD)

}

FrobeniusNorm <- function(G_True, G_Hat) {

F_Norm <- norm(G_True - G_Hat, type = "F")

return(F_Norm)

}

extract_leaf_data <- function(tree, data) {

predictions <- predict(tree, type = "node")

terminal_nodes <- nodeids(tree, terminal = TRUE)

leaf_data <- lapply(terminal_nodes, function(node_id) {

data[predictions == node_id, ]

})

names(leaf_data) <- paste("Node", terminal_nodes, sep = "")

return(leaf_data)

}

### Activate the virtual environment of Python

use_condaenv("my_env", required = TRUE)

py_config()

py_install("ucimlrepo")

ucimlrepo <- import("ucimlrepo")

### Load the empirical dataset

real_data <- ucimlrepo$fetch_ucirepo(name = "Drug Consumption (Quantified)")

### Read covariates

DrugConsumptionFeatures <- real_data$data$features

### Read drug consumption outcome

DrugConsumptionTargets <- real_data$data$targets

### Convert discrete outcome data to continuous

DCTargetsNum <- DrugConsumptionTargets %>% mutate(across(.cols = where(is.character),

~ as.numeric(substring(.x,3,3))))

apply(DCTargetsNum, 2, function(x) summary(factor(x)))

### Delete unnecessary variables

DCTargetsNum <- DCTargetsNum[,-18]

summary(DCTargetsNum)

### Extract two important covariates: nscore (neuroticism) and ss (sensation-seeking)

DCFeatures <- DrugConsumptionFeatures[,c("nscore", "ss")]

DrugConsumptionAll <- cbind(DCFeatures, DCTargetsNum)

### Build the SELF model based on the overall data

SELF_result <- fhc(D = DCTargetsNum, booster = "lm", verbose = TRUE)

Targetname <- colnames(DrugConsumptionTargets)[-18]

FeatureName <- c("nscore", "ss")

DrugConsumptionAll[, Targetname]<- DrugConsumptionAll[, Targetname] %>% mutate(across(.cols = where(is.factor),

~ as.numeric(.x)))

### Identify the impact of covariates using the CTree model

CTree_DrugCsmp <- SELFCTree(

nodevars = DrugConsumptionAll[, Targetname],

splitvars = DrugConsumptionAll[, FeatureName],

model = "correlation",

transform = "cor"

)

### Extract leaf node data from the tree model

leaf_data <- extract_leaf_data(CTree_DrugCsmp, DrugConsumptionAll)

### Build the SELF model based on each leaf node data

SELFList_Drug <- list()

for (p in 1:length(leaf_data)) {

D <- leaf_data[[p]][, Targetname]

SELFList_Drug[[p]] <- SELF::fhc(D = D, booster = "lm")

}

### Descriptive statistics results based on the overall data

plots_list <- list()

df <- DrugConsumptionTargets[,-18]

df <- as.data.frame(lapply(df, function(x) ifelse(x %in% c("CL0", "CL1"), "non-user", "user")))

for (col_name in names(df)) {

col_data <- df[[col_name]]

freq_table <- table(col_data)

freq_df <- data.frame(String = names(freq_table), Frequency = as.numeric(freq_table))

p <- ggplot(freq_df, aes(x = String, y = Frequency)) +

geom_bar(stat = "identity") +

theme_minimal() +

theme(axis.text.x = element_text(angle = 0, hjust = 0)) +

geom_text(aes(label = Frequency), vjust = -0.2, color = "black") +

labs(title = col_name, x = NULL, y = "Frequency")+

ylim(0, 2000)

plots_list[[col_name]] <- p

grid.arrange(grobs = plots_list, ncol = 3, nrow = 6)

}

# Directed acyclic graph result based on overall data

qgraph::qgraph(SELF_result, directed = TRUE, layout = "circle", arrows = TRUE,

edge.color = "black", labels = Targetname,

vsize = 10, esize = 1, repulsion = 0.1)

### Plot the tree structure

ggparty(CTree_DrugCsmp,

terminal_space = 0.5,

add_vars = list(p.value = "$node$info$p.value")) +

geom_edge(size = 1.5) +

geom_edge_label(colour = "grey", size = 6) +

geom_node_label(aes(col = splitvar),

line_list = list(aes(label = paste("Node", id)),

aes(label = splitvar),

aes(label = paste("p =", formatC(p.value, format = "e", digits = 2)))),

line_gpar = list(list(size = 12, col = "black", fontface = "bold"),

list(size = 12),

list(size = 12)),

ids = "inner") +

geom_node_label(aes(label = paste0("Node ", id, ", N = ", nodesize)),

fontface = "bold",

ids = "terminal",

size = 4,

nudge_y = 0.01) +

theme(legend.position = "none")

### Descriptive statistics results of every leaf node data

plots_list <- list()

i = 4

df <- leaf_data[[i]][,3:ncol(leaf_data[[i]])]

prefix <- "CL"

df_with_prefix <- df %>%

mutate(across(everything(), ~paste0(prefix, .)))

df_with_prefix <- as.data.frame(lapply(df_with_prefix,

function(x) ifelse(x %in% c("CL0", "CL1"), "non-user", "user")))

for (col_name in names(df_with_prefix)) {

col_data <- df_with_prefix[[col_name]]

freq_table <- table(col_data)

freq_df <- data.frame(String = names(freq_table), Frequency = as.numeric(freq_table))

p <- ggplot(freq_df, aes(x = String, y = Frequency)) +

geom_bar(stat = "identity") +

theme_minimal() +

theme(axis.text.x = element_text(angle = 0, hjust = 0.5)) +

geom_text(aes(label = Frequency), vjust = -0.2, color = "black") +

labs(title = col_name, x = NULL, y = "Frequency")+

ylim(0, 400)

plots_list[[col_name]] <- p

}

grid.arrange(grobs = plots_list, ncol = 3, nrow = 6)

# Plot DAG results for leaf node data

qgraph::qgraph(SELFList_Drug[[1]], directed = TRUE, layout = "circle", arrows = TRUE,

edge.color = "black", labels = Targetname,

vsize = 10, esize = 1, repulsion = 0.1)

qgraph::qgraph(SELFList_Drug[[2]], directed = TRUE, layout = "circle", arrows = TRUE,

edge.color = "black", labels = Targetname,

vsize = 10, esize = 1, repulsion = 0.1)

qgraph::qgraph(SELFList_Drug[[3]], directed = TRUE, layout = "circle", arrows = TRUE,

edge.color = "black", labels = Targetname,

vsize = 10, esize = 1, repulsion = 0.1)

qgraph::qgraph(SELFList_Drug[[4]], directed = TRUE, layout = "circle", arrows = TRUE,

edge.color = "black", labels = Targetname,

vsize = 10, esize = 1, repulsion = 0.1)

###

i = 3; j =4 # Change the number to calculate the SHD and FNorm

StructuralHammingDistance(G_True = SELFList_Drug[[i]], G_Hat = SELFList_Drug[[j]])

FrobeniusNorm(G_True = SELFList_Drug[[i]], G_Hat = SELFList_Drug[[j]])
